# Favipiravir Analogues as Inhibitors of SARS-CoV-2 RNA-Dependent RNA Polymerase, Combined Quantum Chemical Modeling, Quantitative Structure–Property Relationship, and Molecular Docking Study

**DOI:** 10.3390/molecules29020441

**Published:** 2024-01-16

**Authors:** Magdalena Latosińska, Jolanta Natalia Latosińska

**Affiliations:** Faculty of Physics, Adam Mickiewicz University, Uniwersytetu Poznańskiego 2, 61-614 Poznań, Poland

**Keywords:** favipiravir analogues, interaction patterns, hydrogen bonds, binding modes, COVID-2019, SARS-CoV-2, RNA viruses

## Abstract

Our study was motivated by the urgent need to develop or improve antivirals for effective therapy targeting RNA viruses. We hypothesized that analogues of favipiravir (FVP), an inhibitor of RNA-dependent RNA polymerase (RdRp), could provide more effective nucleic acid recognition and binding processes while reducing side effects such as cardiotoxicity, hepatotoxicity, teratogenicity, and embryotoxicity. We proposed a set of FVP analogues together with their forms of triphosphate as new SARS-CoV-2 RdRp inhibitors. The main aim of our study was to investigate changes in the mechanism and binding capacity resulting from these modifications. Using three different approaches, QTAIM, QSPR, and MD, the differences in the reactivity, toxicity, binding efficiency, and ability to be incorporated by RdRp were assessed. Two new quantum chemical reactivity descriptors, the relative electro-donating and electro-accepting power, were defined and successfully applied. Moreover, a new quantitative method for comparing binding modes was developed based on mathematical metrics and an atypical radar plot. These methods provide deep insight into the set of desirable properties responsible for inhibiting RdRp, allowing ligands to be conveniently screened. The proposed modification of the FVP structure seems to improve its binding ability and enhance the productive mode of binding. In particular, two of the FVP analogues (the trifluoro- and cyano-) bind very strongly to the RNA template, RNA primer, cofactors, and RdRp, and thus may constitute a very good alternative to FVP.

## 1. Introduction

### 1.1. Motivation of the Research

Over the last two centuries, there have been several pandemics/epidemics caused by different human-infective RNA viruses: in 1889–1890 (Asiatic/Russian flu, A subtype H2), in 1918–1920 (Spanish flu, H1N1), in 1957–1958 (Asian flu, H2N2), in 1968–1969 (Hong Kong flu, H3N2), in 1977–1979 (Russian flu, H1N1), since 1981 (AIDS, HIV), in 2009–2010 (Swine flu, H1N1/09), in 2002–2003 (SARS, SARS-CoV), and in 2012–2013 (MERS, MERS-CoV). The emergence of Swine flu 13 highlighted the urgent need for effective antiviral therapy against RNA viruses due to their pandemic nature. The coronavirus disease-2019 (COVID-19), a life-threatening infectious disease caused by the novel severe acute respiratory syndrome coronavirus-2 (SARS-CoV-2) [1,2], which emerged in Hubei Province in Central-Eastern China in December 2019, caused panic and accelerated these efforts. Since the global spread of the COVID-19 pandemic, many different SARS-CoV-2 variants (Alpha, Beta, Gamma, Delta, Epsilon, Etha, Iota, Kappa, Lambda Omicron, Theta, and Zeta) and subvariants, in particular, so-called variants of interests (XBB.1.5, XBB.1.16, EG.5, and JN.1) and variants under monitoring (BA.5, CH.1.1, XBB, and BA.2.86) appeared, which raised concerns about the possibility of prolonging the current pandemic and the occurrence of subsequent ones. Therefore, SARS-CoV-2 variants are monitored on an ongoing basis by the World Health Organization Technical Advisory Group on SARS-CoV-2 Virus Evolution [3].

Three recent pandemics, influenza [4], HIV [5], and SARS-CoV-2 [6], not only have common zoonotic origins but also are caused by RNA viruses (negative-sense single-stranded RNA ssRNA (−) or positive-sense single-stranded RNA ssRNA (+)), belonging to the kingdom *Orthornavirae* (realm *Riboviria*). The HIV group antigens (Gag) and influenza virus matrix (M1/M2) proteins also have even evolved from a common ancestor protein [7]. Two pandemic viruses, influenza and coronavirus, infect the respiratory tract, share similar symptoms, and use surface proteins to infect the host cells [8]. However, influenza uses surface glycoproteins hemagglutinin (HA) and neuraminidase (NA) for infection [9], while SARS-CoV-2 (similarly to MERS-CoV, SARS-CoV-1) uses the spike (S) protein [10]. In the case of HIV-1, the mechanisms of entry are different, but the strategy is similar to the one used by SARS-CoV-2 [11]. All three viruses use the viral RNA polymerase to express their proteins, although only SARS-CoV-2 has a proofreading mechanism [12], which has been considered an argument against the possibility of rapid mutation. Furthermore, in the case of the Ebola virus (Zaire Ebolavirus, EBOV), which is an ssRNA (−) enveloped virus from *Riboviria* [13], the trimeric transmembrane glycoprotein (GP) that plays a key role in infection mediates EBOV attachment and entry into host cells. However, EBOV, which is transmitted through body fluids and causes flu-like symptoms, was the cause of the Ebola hemorrhagic fever epidemic in West Africa in 2013–2016. Fortunately, EBOV does not mutate rapidly, which, contrary to initial fears, reduces the risk of a pandemic. In any case, viruses belonging to the same group of ssRNA viruses with a similar structure are considered potential culprits for the next global pandemic. In light of this, effective therapy targeting RNA viruses is highly desirable.

Most anti-viral drugs used in previous pandemics either inhibit the virion’s M2 ion channel, such as amantadine (Gocovri/Symadine/Symmetrel, 1964) and rimantadine (Flumadine, 1964), or inhibit the viral neuraminidase (e.g., oseltamivir (Tamiflu, 1997), zanamivir (Relenza, 1993), peramivir (Rapivab, 2000), and laninamivir (CS-8958, 2009). They prevent the virus from entering (entry/uncoating phase) and exiting cells (release phase). These treatments have demonstrated some effectiveness; however, there is potential for greater effectiveness with more specific treatment methods. The search for more effective drugs with a broad spectrum of activity and an innovative mode of action was dictated by three main factors: poor performance against new variants, the emergence of resistance to antiviral drugs (e.g., amantadine, rimantadine [14,15,16], and oseltamivir [17,18,19]), and the need to treat previously unknown or untreatable diseases. The most current first-line anti-viral therapies either focus on suppressing the cytokine storm (e.g., tocilizumab, a humanized anti-human IL-6 receptor antibody) or act as broad-spectrum antivirals (e.g., favipiravir, ribavirin, and remdesivir) which inhibit the viral RNA polymerase. The priority is to develop or improve broad-spectrum antivirals for the treatment of epidemic diseases caused by emerging or re-emerging viruses.

### 1.2. Favipiravir–State of the Art

**Figure 1:**
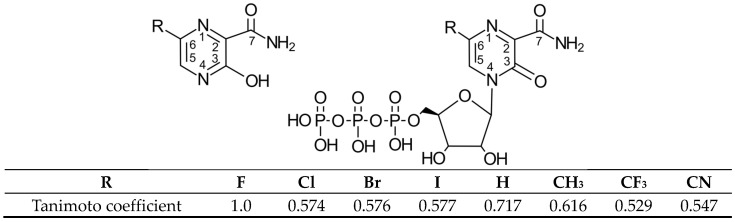
Structural formula of Favipiravir (6-fluoro-3-hydroxypyrazine-2-carboxamide, FVP, R = F) and its analogues R = H, Cl, Br, I, CH_3_, CF_3_ and CN (pro-drug (left), ribofuranosyl-5′-triphosphate active forms (right)) and the Tanimoto coefficient (molecular similarity distance).

Favipiravir (6-fluoro-3-hydroxypyrazine-2-carboxamide, FPV, T-705;), Figure 1, is an active pharmaceutical ingredient discovered by a Japanese company Toyama Co. Ltd. (now Toyama Kagaku Kōgyō, Toyama, Japan), which was approved for treating influenza strains unresponsive to adamantanes or neuraminidase inhibitors [20].

Food and Drug Administration (FDA) phase II and III clinical trials demonstrated the safety of FVP in humans and better efficacy against the influenza virus than oseltamivir [21]. In influenza virus-infected patients treated with FVP, the high barrier to resistance was observed [22]. FVP has been shown to have broad antiviral activity at higher concentrations against many other RNA viruses [23,24,25,26], such as OTV-resistant influenza A, B, and C viruses; as well as flavi-, alpha-, filo-, bunya, and arena-noroviruses [27,28]; Ebola [29]; Lassa virus [30]; West Nile Fever [31]; Zika [32]; Rift Valley fever [33]; Yellow fever [34]; Crimean-Congo hemorrhagic fever (CCHF) [35]; Nipah tick-borne encephalitis [36]; rabies [37]; and Argentine hemorrhagic fever (Junín) [38], including those for which there is currently no antiviral treatment available. After the identification of the new virus, SARS-CoV-2, FVP was among the first medications screened for its effectiveness and safety. Due to its mechanism of action, preclinical results, and high safety in humans, FVP shows promise in treating human diseases caused by various RNA viruses, including SARS-CoV-2. According to the ClinicalTrials.gov database, there are 67 completed, ongoing, or planned clinical trials around the world (Europe, North America, and Australia) assessing the efficacy and safety of FPV in monotherapies and combination therapies in the treatment of COVID-19. (Another 12 are efficacy studies for Influenza (7), Ebola (3), Lassa (1), and CCHF (1).) Currently, FVP is registered as a drug for COVID-19 in China, India, and Russia and is approved in Turkey, Hungary, Serbia, the KSA, Thailand, Egypt, Bangladesh, Pakistan, Jordan, and Saudi Arabia [39,40,41]. Oral doses of FVP have been shown to be safe in outpatients with mild to moderate infections. Furthermore, the reduction in viral load and improvement in radiological and clinical outcomes are significant [24,25]. Compared to Molnupiravir (MOL), Nirmatrelvir (NMVr) [18,19], or Remdesivir (RDV) [42], FVP exhibits reduced potency against SARS-CoV-2 in vitro but similar effectiveness in animal models. 

Certain studies have indicated a potential association between FVP and oxidative stress/cardiotoxicity, [43] hepatotoxicity [44], teratogenicity [45,46,47], and embryotoxicity [48]. However, other anti-viral drugs, including MOL, NMVr, or RDV, also exhibit hepatotoxicity [49], nephrotoxicity [50], and mutagenicity [51]. Inconsistencies in clinical trial results, despite the synergistic effect of combined therapy with Oseltamivir (OSE) [52,53], Ivermectin (IVM) [54], Ribavirin (RBV) [55], Remdesivir (RDV), and Tocilizumab (TOZ) [56] in the fight against H1N1, H3N2, H5N1, and SARS-CoV-2 [57], have caused the use of FVP as a regular drug to remain under investigation. Even RDV, although introduced as a drug for the treatment of COVID-19 after obtaining its first emergency use authorization in May 2020 in the United States [58] and then later in Japan, has faced much criticism. The FDA approved RVD for the treatment of hospitalized patients, and the EU Commission granted conditional authorization, but the European Medicines Agency (EMA) approved it only for patients 12+ suffering from pneumonia who require oxygen supply [59]. The only anti-viral drug approved by the FDA is Pfizer’s Paxlovid^TM^, which is the combination of Nirmatrelvir (NMVR)—SARS-CoV-2 3-CL Mpro inhibitor and Ritonavir (RTV)—protease inhibitor. Thus, its mechanism of action is completely different. However, its use carries many risk factors, including severe liver impairment or liver disease. Recent studies show that SARS-CoV-2 has acquired phenotypic resistance to RDV and Paxlovid^TM^ [60] but not to FVP. Thus, it is highly desirable to seek more suitable FVP alternatives, keeping in mind the issues mentioned above. 

### 1.3. Favipiravir—Unique Mechanism of Action

**Figure 2:**
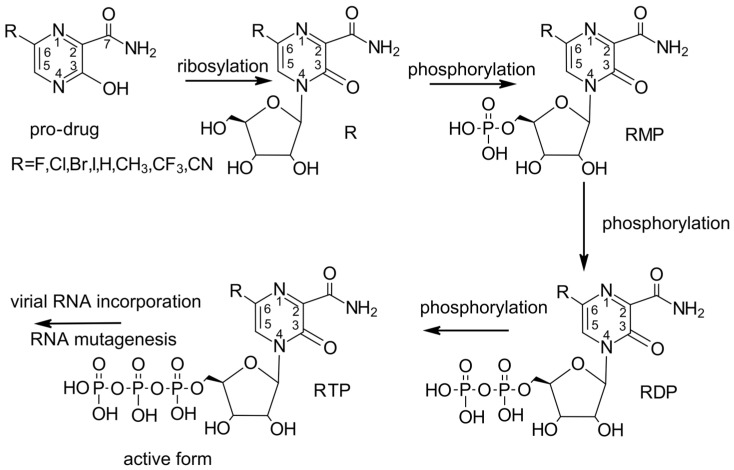
Conversion of favipiravir and its analogues to its various metabolites (a series of ribosylation and phosphorylation steps to form the active triphosphate FVP-RTP).

Due to its relatively simple structure, FVP seems to be easily modifiable while maintaining its unique mode of action. It is therefore an attractive basis for investigating new drugs. However, the exact molecular mechanism behind the broad-spectrum antiviral activity of FVP has not yet been fully elucidated. It is known that FVP, a pro-drug, must be first converted by hypoxanthinguanine phosphoribosyl transferase (HGPRT) to FVP-ribosyl 5′-monophosphate (FVP-RMP) and then metabolized to the FVP-ribofuranosyl-5′-triphosphate (FVP-RTP) by cellular kinases [20,21] to inhibit viral RNA-dependent RNA polymerase (RdRp), as shown in Figure 2.

RdRp, a key enzyme regulating both the replication and transcription of viral RNA, is a component of the so-called minimal core of viral RNA replication. FVP-RTP acts as a purine nucleoside analogue, which pairs with either cytosine or uracil. It competitively inhibits the viral RdRp polymerase substrate, leading to chain termination [61] and ultimately inhibits the transcription and replication of the viral genome RNA. Another possible mechanism of its action is lethal mutagenesis, resulting in an increased frequency of, primarily, guanosine to adenine (G→A) and, secondarily, C→U mutations, which produce non-infectious progeny during replication [62]. In this case, the therapeutic effect of FPV will result from the accumulation of mutations in the replicated RNA of nascent viruses, which leads to a cascade of mistakes, the so-called “error catastrophe”, and loss of the virus’s ability to reproduce. The malfunction of RdRP in the presence of FPV depends on the intracellular phosphorylation of the drug to its active form (FPV triphosphate), a false nucleoside that is built by the viral RdRP into the nascent viral RNA, resulting in a “defective”, mutated RNA. Since RdRp structure and function are conserved among RNA viruses and no RdRp homolog has been found in human cells, its selection as a target is highly advantageous for the development of broad-spectrum antivirals. Furthermore, it was observed that the conserved active site of RdRp does not mutate as easily as other targets, such as the Spike protein. However, FVP is considered a poor substrate for HGPRT, and the requirement for its conversion to the active metabolite RTP is, in fact, a limiting factor for its antiviral activity [63]. Viral resistance to FVP may result from depletion or a lack of HGPRT [44]. In HGPRT-deficient cells, FVP is completely devoid of antiviral activity. To overcome the inadequate activation of FVP, the utilization of ribonucleoside pro-drugs, as well as di- and triphosphate analogues, seems to be a viable solution, which increases the biodistribution and therapeutic efficacy of FVP. 

### 1.4. FVP Analogues—A Research Hypothesis and Concept

The motivation for our study is the hypothesis that new FVP analogues may provide more efficient nucleic acid recognition and binding processes while reducing side effects. Based on our previous observations from the analysis of FVP binding modes in solid, pre-catalytic, and active forms [64], we proposed a set of the FVP analogues (pro-drugs) along with their triphosphate forms as novel SARS-CoV-2 RdRp inhibitors. The pyrazine heterocyclic ring and three functional groups (halogen/CH_3_/CF_3_, hydroxide, and amide) in the FVP analogues allow them to engage in weak non-covalent interactions (e.g., hydrogen-bonds, van der Waals, steric, and stacking). The presence of the three donor (amide and hydroxide) or five/eight acceptor atoms (i.e., two aromatic ring nitrogens, oxygens of amide and hydroxide, and halogen/CF_3_/CN) in one molecule facilitates the formation of hydrogen bonds in the parental FVP. According to the Etter rule [65], FVP analogues can theoretically realize up to twenty-four different types of hydrogen bonds, which makes them highly appealing for pharmaceutical purposes. Enzymatic conversion to the active ribofuranose-5′-triphosphate (RTP) form leads to an increase in the number of oxygen atoms while facilitating the establishment of hydrogen bonds. However, both of these factors can be modified by altering the substituents at the C(6) position of the pyridazine ring. The key question is whether and to what extent alteration of the molecular structure via substituents will help in the effective recognition of FPV analogues, their incorporation into the RNA strand, and their binding to the RdRp. The effective screening of the halogenated and non-halogenated (R = I, Br, Cl, CF_3_, H, CH_3_, CF_3,_ or CN) analogues of FVP-RTP is the objective of the current research. Using the Quantum Theory of Atoms in Molecules (QTAIM), Quantitative Structure–Property Relationship (QSPR), and Molecular Docking (MD) approaches, the differences in their reactivity, toxicity, binding efficiency, and ability to be incorporated by SARS-CoV-2 RdRp were assessed. These methods, supplemented by new global indices describing relative reactivity and new quantitative methods for the estimation and visualization of the differences in the binding modes of individual analogues with RdRp, provide insight into the set of desirable characteristics responsible for the inhibition of the SARS-CoV-2 RdRp. Molecular docking studies demonstrate the high binding affinity of the FPV analogues to SARS-CoV-2, indicating their potency as antiviral drugs against COVID-19. The proposed modification of the FVP structure seems to improve its binding ability to SARS-CoV-2 RdRp and enhance productive binding modes. If sufficient efficacy in inhibiting viral replication in cell culture is established, they could be explored as potential drugs against COVID-19. Our method for quantifying differences in binding mode holds promise for guiding future research on new anti-SARS-CoV-2 agents.

## 2. Results and Discussion

### 2.1. Characteristics of the Candidate Ligands

#### 2.1.1. Physicochemical Profile (ADMET) and Key Pharmacokinetic Parameters

FVP analogues (Figure 1, R = I, Br, Cl, CF_3_, H, CH_3,_ CF_3_, or CN) were selected as candidate ligands for SARS-CoV-2 RdRp and potential pro-drugs. The physicochemical profile parameters that describe the pharmacokinetic behavior of the candidate ligands and known drugs (MOL, RVD, and the only registered drug, Paxlovid^TM^, components: NMVr and RTV) have been evaluated in Table 1 and Table 2.

The molecular weights of the ligands (MW) ranged from 139.11 to 265.01 g/mol and did not exceed 500 g/mol. According to Swiss ADME [66], the predicted consensus LogP (lipophilicity) for the ligands ranged from −0.83 to 0.52. The CN analogue, like FVP, showed low and negative lipophilicity, while the CF_3_ analogue showed high and positive lipophilicity (closer to optimal for drugs). The substitution of –F with –CF_3_ increases the hydrophobicity (lipophilicity) of the ligand. Thus, the alteration of lipophilicity has the potential to modify hydrophobic targets. Indeed, in medicinal chemistry, –CF_3_ is often used as a substituent due to its strong electron-withdrawing nature, poor polarizability, and broad hydrophobic domain [67] (e.g., Tecovirimat [68], Doravirine [69], and Tipranavir [70]). However, ADMETlab 2.0 [71], which uses a different model, predicts negative LogP for both above-mentioned ligands. The predicted water solubility index (Solubility, SILICOS-IT) suggests that all the candidate ligands, like FVP, should be highly water soluble. Moreover, none of them showed lead-likeness violations and no violation of drug-like rules (Lipinski, Egan, or Veber). The predicted synthetic accessibility score (SAS) for the ligands ranges from 1.73 to 2.43, indicating the ease of synthesis of these compounds.

All the ligands showed high gastrointestinal absorption (GI), a key parameter in assessing the in vivo performance of an orally administered drug formulation. Abbot’s Bioavailability score for all ligands placed them within the 55% probability class. The topological polar surface area (TPSA), which describes the passive molecular transport across the membranes, is 88.84, except for the CN analogue, which showed a significantly higher TPSA of 112.63. However, since TPSA does not exceed 140, it is still an optimal level. Overall, the physicochemical profile of the candidate is better than those of MOL, RDV, NMVr, and RTV. The candidate ligands show no Pan Assay of Interference Structures (PAINS) or Structural (BRENK) alerts. None of the ligands are expected to cross the blood–brain barrier (BBB), inhibit cytochrome P450 (CYP) isoforms or have a potential to be a substrate of multidrug resistance protein (permeability glycoprotein, P-gp). The risk scores for carcinogenicity or genotoxic mutagenicity are very low for all of them, while the risk scores for hepatotoxicity (H-HT) and drug-induced liver injury (DILI) in humans are high. However, the CF_3_ analogue showed two times lower hepatotoxicity than FVP. The probability of genetic toxicity (*Salmonella typhimurium* reverse mutation assay, i.e., AMES assay) and the probability of rat acute oral toxicity (LD_50_) are relatively low for the CN and CH_3_ derivatives. Overall, the AMDE toxic profile for candidate ligands is non-inferior to FVP and better than MOL and RVD in terms of TPSA, genotoxic mutagenicity, genetic toxicity, H-HT, and DILI. The NVMr shows smaller toxicity, but it is combined with highly toxic RTV.

However, there is no set of ideal pharmacokinetic parameters that a given drug candidate should exhibit, as it depends on the specific requirements of the target. Therefore, our objective is to identify drugs that possess an optimal pharmacological profile, achieving the desired biological activity with minimal side effects (the last rows of Table 1 and Table 2 show the values that can be considered optimal).

#### 2.1.2. Reactivity Profile (Quantitative Structure–Property Relationships)

Each ligand (potential pro-drug) and its active form, ribofuranosyl-5′-triphosphate (RTP), were constructed and optimized at the B3LYP/6–311G(d,p) level of theory. The enol tautomer (with intramolecular OH⋯O hydrogen bond), although more stable than the keto tautomer in solids and solutions, is further excluded by ribofuranosyl substitution. The planar conformation of the pyrazine moiety in active forms is maintained and supported by the intramolecular NH⋯O hydrogen bond in all FVP analogues, regardless of the type of substituent. The optimized molecular geometries served as the initial configurations for further QSPR and MD studies.

Determining the impact of molecular structure on reactivity is essential when designing the ligands with desired properties. A powerful tool for describing the chemical reactivity of the ligands based on their structure is the frontier molecular orbital (FMO) theory, which conceptualizes chemical bonding and reactivity in terms of the interactions between frontier orbitals. The highest occupied molecular orbital (HOMO) and lowest unoccupied molecular orbital (LUMO) are very useful for assessing the chemical reactivity of molecules. LUMO accepts electrons, and its energy corresponds to an electron affinity (EA), while HOMO donates electrons, and its energy is related to ionization potential (IP). Low IP and high EA correspond to high nucleophilic and high electrophilic properties, respectively. Therefore, the HOMO-LUMO gap is useful for predicting charge transport and ligand. Theoretical global indices of ligand reactivity, such as absolute electronegativity, χ; absolute hardness, η; electrophilicity index (reactivity), ω; softness, S; electro-donating power, ω^−^; electro-accepting power ω^+^; net electrophilicity, Δω; and a maximum number of electrons transferred in a chemical reaction, ΔN_max_, provide further details regarding the reactivity of the ligands. The HOMO, LUMO, and global reactivity indices for the ligands were evaluated at MP2/6-311G(d,p) and M062X/6-311G(d,p) levels of the theory (in the gas phase—single molecule and aqueous solution) in Table 3 and Table 4.

Comparing reactivity parameters makes sense in relation to groups of structurally similar compounds; therefore, analyzing MOL, RVD, NVMr, or RTV will not bring anything in this respect.

The ligands studied can be ordered according to the decreasing HOMO-LUMO gap as follows: CF_3_ > H > CN > F > CH_3_ > Cl > Br > I. The results show that regardless of the phase (gas or aqueous solution), the CF_3_ and I analogues have the highest and lowest stability, respectively. The CF_3_ and CN analogues, which have a higher HOMO-LUMO energy gap, are more stable and are therefore chemically harder than the other ligands. However, each of these ligands is slightly less stable in aqueous solutions than in the gas phase.

The ordering of the ligands according to decreasing absolute electronegativity, χ, a measure of the ligand’s ability to attract electrons to itself, is correlated with the electronegativity of the halogen: CH_3_ < H < I < Br < Cl < F < CN < CF_3_. The CF_3_ group has a significantly strong electronegativity, typically intermediate between that of F and Cl, while the CN bond is strongly polarized toward nitrogen and more electronegative than Cl. Surprisingly, among the FVP analogues, the CF_3_ derivative is more electronegative than the CN derivative. The ordering of the ligands according to decreasing global hardness, a measure of the ligands’ resistance to change its electronic configuration, is as follows: CF_3_ > H > CN > F > CH_3_ > Cl > Br > I. The very high value of the absolute hardness for the CF_3_ derivative indicates its high degree of stability and low reactivity. The χ value describes the tendency to donate/accept electrons, while η measures the ease with which this can occur, which for CF_3_, are high and low, respectively.

The most important descriptor measuring electrophilic power (capacity of an electrophile to accept the maximal number of electrons in a neighboring reservoir of electron pool) is the global electrophilicity index, ω. Its values for the ligands are in the range of 1.318–1.885 eV and 1.323–1.670 eV in the gas phase and aqueous solution, respectively, as can be seen in Table 1. The parameter ω, which actually measures the reactivity of the ligand, revealed the following trend: CN > CF_3_ > F > Cl > Br > I > H > CH_3_. Therefore, reducing the inductive electron-withdrawing effect (F > Cl > Br > I) and electron-donating effect via resonance (F > Cl > Br > I) leads to a decrease in electrophilic activation (ω = 1.676, 1.648, 1.606, 1.592 eV for F, Cl, Br, and I, respectively). Very high reactivity values describing the system’s tendency to acquire electrons from the environment are observed for the CN and CF_3_ derivatives_,_ while very low values for the H and CH_3_ derivatives. Thus, the CN and CF_3_ analogues seem most promising because highly electrophilic reagents lead to low substrate selectivity, which means they can inhibit a wide range of RdRps, not just SARS-CoV-2. Both analogues also have the highest local electro-donating power, ω^+^, electro-accepting power, ω^−^, and overall electrophilicity, Δω. A larger ω^+^ value for CN corresponds to its better ability to accept charge, whereas a smaller ω^−^ value for CN makes this ligand a better electron donor. However, the unusually low-lying LUMO level for the CN and CF_3_ analogues suggests their easy participation in molecular reactions with nucleophiles, and the low-lying HOMO level for the CN and CF_3_ analogues suggests their easy participation in molecular reactions with electrophiles. LUMO is even slightly lower, while HOMO is slightly higher in the aqueous solution than in the gas phase, while ω^+^ and ω^−^ are higher in the gas phase.

Two new reactivity descriptors, the so-called relative electron donation power, *R^+^*, and the relative electro-accepting power, *R^−^*, have been defined by us.

The relative electro-donating power, *R^+^*, is the quotient of the electro-donating power of the tested ligand and the reference ligand:

(1) $$R^{+}=\frac{\omega_{ligand}^{+}}{\omega_{reference\;ligand}^{+}}$$

Similarly, the relative electro-accepting power is the quotient of the electro-accepting power of the tested ligand and the reference ligand:

(2) $$R^{-}=\frac{\omega_{ligand}^{-}}{\omega_{reference\;ligand}^{-}}$$

Both parameters, *R^+^* and *R^−^*, describe the ability of the ligands to accept and donate charge, as can be seen in Table 3 and Table 4.

The orderings of the ligands in descending order of *R^+^* and *R^-^* are as follows: 

CN > CF_3_ > F > Cl > I > Br > H > CH_3_ and CN > CF_3_ > F > Cl > Br > I > H > CH_3_ (the gas phase)

and

CN > CF_3_ > F > Cl > Br > I > H > CH_3_ and CF_3_ > CN > F > Cl > Br > I > H > CH_3_ (aqueous solution).

**Figure 3:**
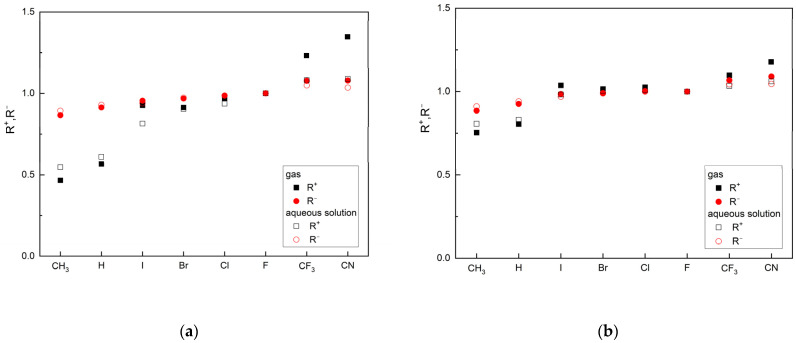
The ligand’s ability to accept (*R^+^*) and donate (*R^−^*) charge in relation to FVP: (**a**) MP2/6-311G(d,p) and (**b**) M062X/6-311G(d,p) (the ordering of the substituents according to group electronegativity).

Both parameters, *R^+^* and *R^−^*, allow for the classification of ligands based on the reference ligand, as can be seen in Figure 3. 

The *R^−^* values in the gas phase and the aqueous solution are similar, while the R^+^ values differ significantly. Based on these parameters, the two ligands CN and CF_3_ are better as both acceptors and donors than FVP. These features are maintained in the aqueous solution (pH = 7, i.e., close to body pH). Moreover, the conclusions remain the same regardless of the calculation method used. However, the MP2 function appears to be more sensitive to changes in reactivity, as shown in Figure 3.

The degree of interaction, DOI [65], calculated for the most promising ligands in Table 5 confirms the observations regarding the differences between the two analogues CN and CF_3_.

The DOI characterizes the strength of an atom’s attachment to its molecular neighborhood, i.e., the degree of electron density sharing between the atom and its surroundings. The highest DOI was obtained for the acceptors –N(4) (39.26% in FVP, 37.964 for CN, and 37.963 for CF_3_), followed by –C(3) (20.942% for FVP, 20.651% for CN, and 20.300% for CF_3_), and –C(2) (22.819% for FVP, 22.352% for CN, and 22.349% for CF_3_). The lowest DOI was obtained for –NH_2_ (1.053% for FVP, 1.18% for CN, and 1.024% for CF_3_), –OH (2.626% for FVP, 2.449% for CN, and 2.571% for CF_3_) acting as a donor, and =O (0.141 for FVP, 0.143 for CN, and 0.156 for CF_3_%). The very low DOI for the –NH_2_ group is maintained regardless of the ligand type, making it highly suitable for binding ligands to the RNA strand. Based on the DOI parameters, the CF_3_ ligand appears more attractive than CN. After ribofuranosyl substitution, any R substituent loses the ability to share electron density with its surroundings in favor of RTP, and therefore, RTP plays a dominant role in the formation of bindings with the protein.

### 2.2. Characteristic of RNA-Directed RNA Polymerase

The genomic arrangement of SARS-CoV-2 is primarily composed of 4 structural proteins, nucleocapsid protein (N), spike protein(S), envelope protein (E), and membrane protein (M); 16 non-structural (NSPs); and 9 accessories (ORFs). Therefore, the SARS-CoV-2 virus consists of many proteins that can mutate rapidly. Two-thirds of the viral genome is occupied by the replicase gene referred to as two Open Reading Frames (ORFs), ORF 1a and ORF1ab, which encode the non-structural proteins (NSPs), the so-called pp1a and pp1ab polyproteins, respectively. The non-structural protein pp1ab includes RNA-directed RNA polymerase (RdRp), so-called nsp12 (chain 4393–5324). RdRp is a core component of viral replication and transcription [26] and exhibits significant catalytic activity, but only with the help of other cofactors: nsp7 and nsp8 [26,27]. Thus, nsp12-nsp7-nsp8 is defined as the minimal core component for viral RNA replication. It has been observed that the conserved active site of RdRp does not mutate as easily as other targets, such as the S protein.

The palm subdomain of SARS-CoV-2 RdRp (residues 585–625 and 680–807) forms the catalytic core of the polymerase, which contains the four highly conserved motifs (A–D). The principal target for SARS-CoV-2 is the active site of the RdRp polymerase, which is formed by two catalytic motifs: A, composed of the residues from 611 to 626, and C, containing residues from 753 to 767 [72]. In general, different RdRp polymerase inhibitors bind more or less strongly with the following residues: ASP760, ASP761, GLY616, TRP617, ASP618, TYR619, PRO620, LYS621, CYS622, LEU758, SER759, ALA762, ALA797, LYS798, CYS799, TRP800, HIS810, GLU811, PHE812, CYS813, SER814, and GLN815 [73,74]. Three hydrophilic and polar residues, ASP618, ASP760, and ASP761, play a key role in SARS-CoV-2 RdRp inhibition [74]. ASP618 is the most conserved residue in viral RdRp and, together with two strictly conserved residues, ASP760 and ASP761, is responsible for the formation of the RdRp catalytic center. ASP623 is involved in a hydrogen bond with the 2′-OH group of the nucleoside triphosphate and therefore appears to be important in sugar selection. The neutral SER759 residue is involved in the positioning of the priming nucleotide [75,76], while the hydrophilic and polar LYS798 stabilizes the RdRp core [77]. The multi-subunit RdRp binds nucleotide triphosphate substrates that enter the main enzyme channel via a hydrophilic cluster formed by the polar residues LYS545, ARG553, and ARG555. When the substrate enters the active site of the enzyme, the complex is formed.

### 2.3. Binding Modes of the Native and Candidate Ligands to RdRp in Different States

The procedure used to dock FVP-RTP analogues (Figure 1, R = I, Br, Cl, CF_3_, H, CH_3,_ CF_3,_ or CN) to the SARS-CoV-2 RdRp was nearly identical to that previously described [64,78,79]. The binding site (cavity) was identified, and the search space was defined as a subset region of about 9.0–15.0 Å.

Knowing that FVP-RTP can effectively mimic either guanosine or adenosine and bind to cytosine or uracil [66,80], both potential possible methods to bind to the SARS-CoV RdRp were explored. This may help resolve the source of bias in the spectrum of mutations induced by FVP, which is likely to be competition with adenosine and guanosine during nucleotide incorporation. Furthermore, the differences between desirable/productive and undesirable/unproductive binding modes were analyzed. To evaluate the quality of the docking process, we performed a redocking task. In each case, the actual ligand was removed from the parental structure and redocked in its own binding site. The redocking protocol was considered successful when the root-mean-square deviation (RMSD) of the pose relative to its conformation in the parental structure did not exceed 3 Å. The binding mode of the native ligand to the RdRp was described in detail. Then, in the same way, the new ligand was docked into the rigid protein structure, and its binding mode was characterized.

#### 2.3.1. Pre-Catalytic State—Productive Mode I (Binding to Cytosine and Stacking to Adenosine)

##### Binding Mode of the Native FVP-RTP Ligand to RdRp

**Figure 4:**
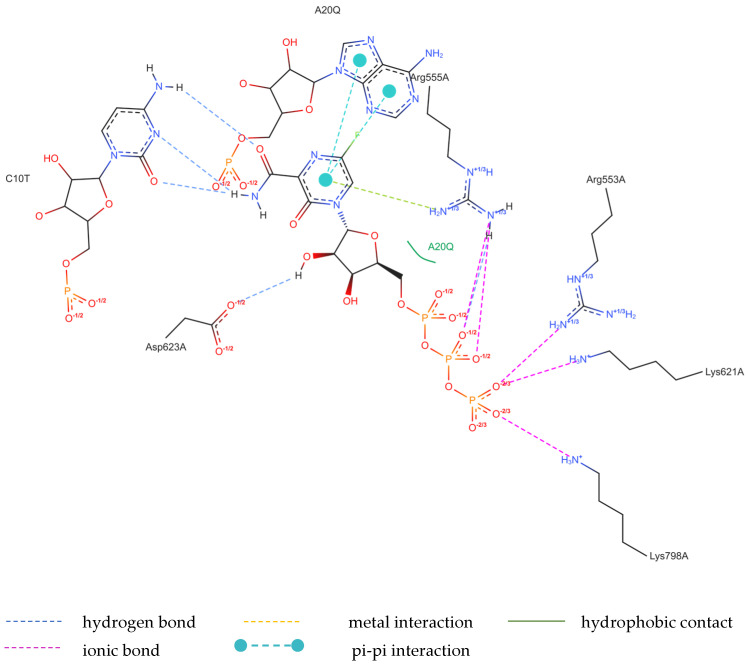
The binding mode of FVP-RTP to RdRp in the 7CTT [81] complex.

The structure of the replicating polymerase complex of SARS-CoV-2 with FVP-RTP in the pre-catalytic state (7CTT) [81] was retrieved from the PDB database. The pocket containing FVP-RTP has a surface area of 967.55 Å^2^ and a volume of 998.91 Å^3^, which is thus a surface/volume ratio of 0.97. Its hydrophobicity is 0.58. In this pre-catalytic state, one FVP-RTP molecule is incorporated into the RNA primer strand and forms a base-stacking interaction with adenosine in this strand. The conformation of the amide group in FVP-RTP is stabilized by the intramolecular N–H⋯O hydrogen bond of 2.612 Å (closing 6-member ring), which causes it to resemble guanine and facilitate π⋯π stacking. FVP-RTP is also involved in strong hydrogen bonds (two N–H⋯O of 2.4 Å and 3.2 Å and one N–H⋯N of 2.74 Å) with the cytosine from the RNA template strand. Furthermore, FVP-RTP binds to at least nine RdRp residues and one cofactor (magnesium Mg^2+^ ion) via various non-covalent interactions, as shown in Table 6 and Table 7 and Figure 4.

Non-covalent interactions include one N–H⋯O of 2.99 Å and two O–H⋯O hydrogen bonds of 2.44 Å and 3.38 Å binding FVP-RTP to serine SER682, and plenty of O–H⋯O and N–H⋯O hydrogen bonds between ribosyl and phosphate of FVP-RTP, and the neighboring residues, as shown in Table 1. FVP-RTP is also coordinated with ARG555 via π-cation interaction, with LYS545, which accepts hydrogen bonds, and with ARG555, LYS798, LYS621, and ARG553 via salt bridges. The interaction of FVP-RTP with Mg^2+^ is weak because it is long-range (of 6.94 Å). Fluorine participates in two F⋯O contacts of 4.957 Å and 4.691 Å and supports a C–H⋯O hydrogen bond of 2.694 Å to maintain the specific conformation of RTP. Moreover, the F⋯N contact of 4.151 Å supports the π⋯π stacking. This all adheres to the typical pattern of fluoride’s role in drug structures, specifically its impact on conformation. The binding mode of the candidate ligands should be consistent with that described above.

##### Binding Mode of the Candidate Ligands to RdRp

Docking Results

**Figure 5:**
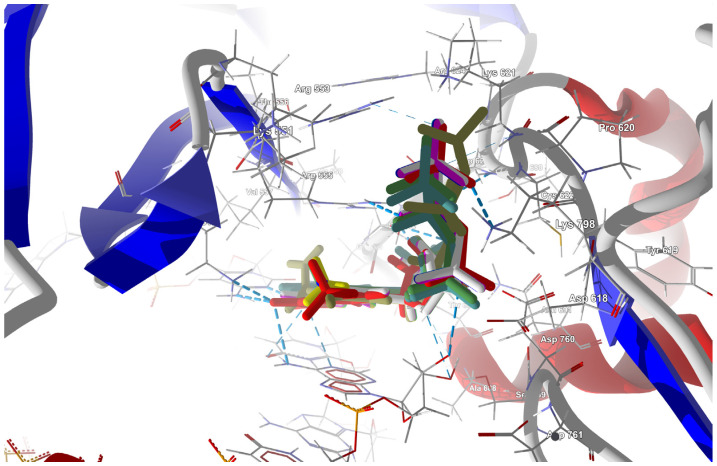
The docked poses of the ligands in the RdRp binding site. The protein backbone is represented as a cartoon, the binding cavity residues are shown as thin sticks, and the docked ligands are shown as color sticks (FVP in cyan, CN in yellow, and CF_3_ in red).

Triphosphorylated forms of the candidate ligands (with the F replaced by I, Br, Cl, H, CH_3_, CF_3_, or CN at the C(6) position) were prepared and docked to the binding site in RdRp that had been previously prepared by correcting protonation and atomic hybridization. The docking results are summarized in Table 8, and the best poses that led to the stabilization of the complex with the highest binding/docking score are shown in Figure 5.

The ordering of the ligands by descending docking score is as follows:

CN > CF_3_ > CH_3_ > Cl > Br > I > H > F.

As shown in Table 6, the total protein–ligand binding energy increased by 20–30% compared to the actual ligand, FVR-RTP. The docking score and total protein–ligand binding energy are highest for the CN analogue, followed by CF_3_ and Cl. According to decreasing total binding energy, the ligands can be ordered as follows:

CN > CF_3_ > Cl > I > CH_3_ > Br > H > F.

The sum of the binding energies of the ligand to the cofactor, RNA Template, and RNA primer is the highest for the CF_3_ analogue, followed by CN and Cl. Protein–ligand hydrogen bonds are the strongest for the CF_3_ analogue, followed by CN and Br. The orderings according to different energy characteristics, as presented in Table 9, show similar trends, i.e., the substitution of F to CF_3_ and CN leads to the most significant changes.

The ordering of the ligands according to the decreasing binding affinity is as follows:

CF_3_ > CN > F > H > Cl ≅ Br > I > CH_3_

**Figure 6:**
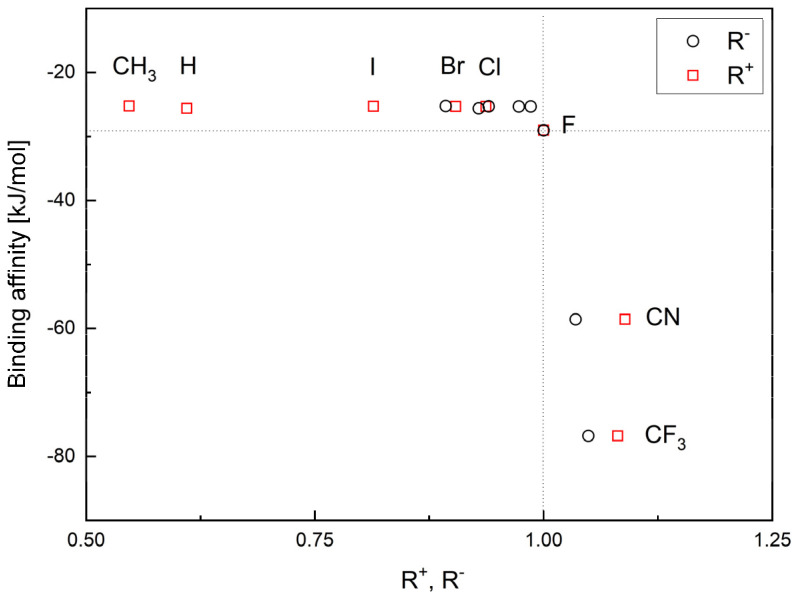
The binding affinity versus ligand’s ability to accept (*R^+^*) and donate (*R^−^*) charge.

The binding affinity is strongly but non-linearly correlated with the relative reactivity power R^+^ and R^−^, Figure 6.

The highest values of *R*^+^ and *R*^−^ correspond to the strongest binding affinity. Thus, the CF_3_ and CN analogues indeed seem the most promising.

In-Depth Analysis of the Binding Mode

The in-depth inspection of the binding mode reveal that all FVP-RTP analogues bind to the same set of the RdRp residues (76 in total), RNA primer, RNA template, and one cofactor (Mg^2+^ ion), although via various non-covalent interactions. In this sense, the binding mode remains consistent among all analogues, while there are differences in the strength and nature of the interactions.

The binding mode of each ligand can be treated as a specific kind of “binding fingerprint”. Global differences in the binding modes of specific ligands relative to the actual ligand FVP-RTP (the entire complex, only protein residues, only cofactors, RNA template, and RNA primer) can be compared using different mathematical metrics (Euclidean or Manhattan) or simple summations, as shown in Table 10 and Table 11. Manhattan distance, which measures distance by pairwise aggregating the absolute difference between each variable, seems the most intuitive for relating the binding mode differences. Euclidean distance, which uses the squared difference in each variable, seems less convenient as it over-optimizes the result. Simple summing (additive method) shows only the balance of contributions.

Although each ligand interacts with as many as 76 residues of RdRp, most of these interactions are of minor importance, as shown in Table 8 and Table 9 and Figure S1. The CF_3_ and CN derivatives show the most significant differences in their binding modes compared to FVP, as Table 6, Table 8 and Table 9 show. Furthermore, these differences arise from different factors. While CN substitution modifies bindings to the RdRp residues, primarily those near the active site, CF_3_ significantly modifies interactions with the co-factors, RNA template, and RNA primer. Moreover, the docking results suggest that among the candidate ligands, the CF_3_ analogue should bind most strongly to both the RdRp, as well as the cofactor, RNA template, and RNA primer, and thus may be a very good alternative to FVP. Although the CN derivative has the highest total binding affinity, as shown in Table 6, it binds relatively weakly to the RNA template due to the unfavorable conformation of its amide group (tilted relative to the plane of the pyridazine ring).

Detailed insight into the binding mode using Ligplot+ [82,83] reveals a set of hydrogen bonds binding the ligands with ARG555, SER682, LYS98, LYS621, and LYS545 and hydrophobic interactions binding the ligands with ASP760, THR687, ASP623, VAL557, ASP618, adenosine, and uracil, as shown in Figure S2, which further stabilizes the protein–ligand complex. It should be noted that Ligplot+ suggests a similar set of hydrophobic interactions for all considered ligands binding to the RdRp. However, ASP623 forms NH⋯O or OH⋯O hydrogen bonds only with the CF_3_ and CN analogues and is therefore not recognized as hydrophobic, as shown in Figure S2 (middle and right).

**Figure 7:**
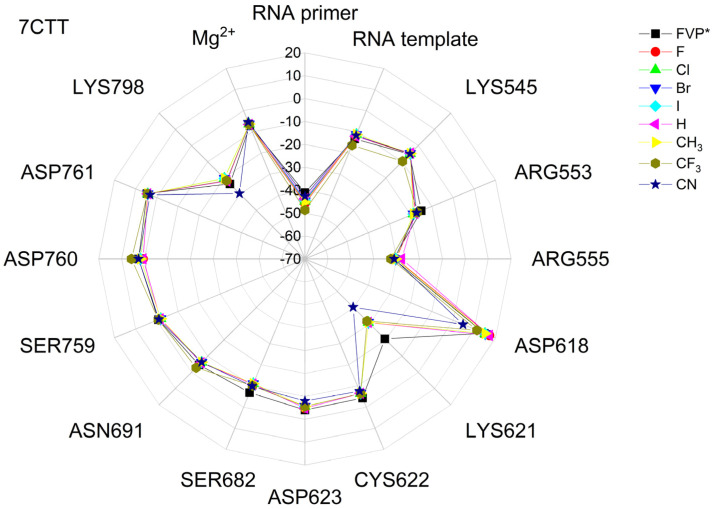
The comparison of the binding energies of FVR-RTP analogues with the RdRp split to active site residues, RNA primer, RNA template, and cofactor. (FVP*—native ligand).

The binding energies of the FVP-RTP analogues to the RdRp residues, RNA primer, RNA template, and cofactor (7CTT [81] target) are summarized in Table 12, and a radar plot comparing these data is shown in Figure 7.

As shown in Table 12 and Figure 7, the replacement of the F at C(6) with I, Br, Cl, CF_3_, H, CH_3,_ CF_3,_ and CN leads primarily to the changes in the binding strength of the ligand to the RNA primer, LYS545, ARG553, ARG555, ASP618, LYS621, CYS622, ASP623, SER682, ASN691, SER759, ASP760, ASP761, and LYS798. Moreover, the interactions of all FVP-RTP analogues with the conserved residue ASP618 and critical residues SER759, ASP760, and ASP761 are primarily electrostatic and repulsive; the bindings to the polar and hydrophilic LYS798, LYS621, SER682, ASP623, and ASN691 are strong and attractive, while their bindings to the hydrophilic cluster formed by LYS545, ARG553, and ARG555 are strong and mainly ionic (salt bridges). The bindings to the Mg^2+^ and Zn^2+^ cofactors are relatively weak, and the latter is negligible. However, the allocation of the binding energy between the individual residues is not uniform, Figure 7. Moreover, some residues, such as LYS798, LYS621, and ASP618, are highly sensitive to the type of ligand.

Regardless of whether only residues near the active site or all of them are taken into account, the most significant changes occur when F is replaced by CF_3_ or CN, as shown in Table 6, Table 8, Table 9 and Table 10. The most promising CF_3_ analogue interacts with the conserved residue ASP618 (11.267 kcal/mol) and three critical residues neutral SER759 (−0.752 kcal/mol), polar ASP761 (4.363 kcal/mol), and ASP760 (5.699 kcal/mol); these interactions are primarily electrostatic and repulsive in nature. Its bindings with LYS798 (−21.657 kcal/mol), LYS621 (−31.682 kcal/mol), SER682 (−10.147 kcal/mol), ASP623 (−5.553 kcal/mol), and ASN691 (−2.873 kcal/mol) are significantly stronger than the other ligands. The binding of the CF_3_ analogue to the hydrophilic cluster residues LYS545 (−9.728 kcal/mol), ARG553 (−16.868 kcal/mol), and ARG555 (−32.469 kcal/mol) is very strong, but its binding to the Mg^2+^ cofactor is relatively weak: only of −6.464 kcal/mol. Moreover, its binding to the RNA template and primer is the strongest among the candidate ligands (−48.652 and −16.309 kcal/mol for the RNA primer and template, respectively). The CN analogue binds to the RNA template and RNA primer less strongly than the CF_3_ analogue, but its binding to LYS621 and LYS798 is much stronger. The cosine distance in Table 10 shows the relatively low similarity of the CF_3_ and CN analogues to the FVP-RTP in terms of binding mode when considering the active site residues, RNA primer, RNA template, and cofactor and even slightly smaller when considering only active site residues. This effect is noticeable in all analogues, but it is noteworthy only in the CN and CF_3_ analogues. Importantly, the discrepancies in molecular fingerprints (Tanimoto distances, Figure 1) do not correspond to the discrepancies in the binding modes. Thus, the alteration in the binding mode resulting from a mere substitution of a group is complex and multifactorial.

Binding Mode Visualization

**Figure 8:**
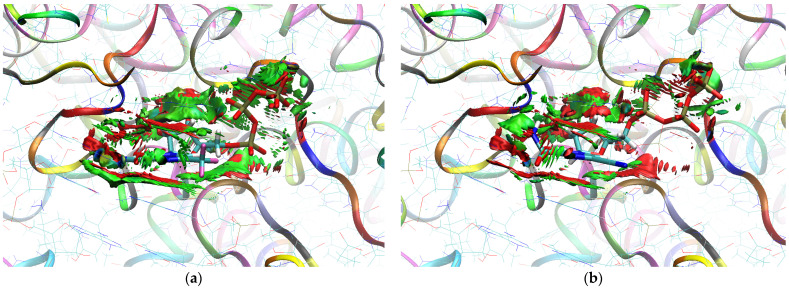
The overlap of the isosurfaces of RDG (isovalue 0.5a.u.) with sign(λ_2_)ρ_BCP_ mapped over the surface for (**a**) FVP (red) and the CF_3_ derivative (green) and (**b**) FVP (red) and the CN derivative (green). (Pre-Catalytic State—Productive Mode I).

The sign[λ_2_(r)]ρ(r) surface mapped to the reduced density gradient, RDG(r), isosurface in the red–green–blue scheme for the RdRp-ligand complex visualizes the binding mode and reveals the nature of the non-covalent interactions between the RdRp and the ligands, as can be seen in Figure 8.

The overlap of the isosurfaces of RDG with sign(λ_2_)ρ_BCP_ mapped over the surface shows the differences in binding mode for the candidate and actual ligand. Weak van der Waals-type interactions clearly dominate, which is indicated by the green color of the surface for each ligand. The large and nearly flat green area above the six-membered ring of the FVP ring proves π⋯π stacking between the ligands and adenosine. The N–H⋯O hydrogen bond linking the ligands to the RNA strand is clearly visible and depicted by a small light cyan disc-shaped area near =O and -NH_2_. The CF_3_ analogue binds more strongly to RdRp than the CN analogue, as confirmed by the significantly larger blue-green surfaces depicting attractive interactions, as shown in Figure 8.

#### 2.3.2. Pre-Catalytic State—Productive Mode II (Binding to Uracil and Stacking to Guanosine)

##### Binding of the Native RVD Ligand to RdRp

The structure of the replicating polymerase complex of SARS-CoV-2 in the pre-catalytic state bound to RVD (7UO4) [80] was retrieved from the PDB database. The pocket containing RDV has a surface area of 1364.91 Å^2^, a volume of 1364.99 Å^3^, and a surface/volume ratio of 1.0. Its hydrophobicity factor is slightly higher and equal to 0.61. The backbone of 7UO4 [80], which holds the protein together and gives it its tertiary structure, differs from 7CTT [81] but only by 0.475% (backbone residues) and 1.817% (all residues). In this form, one RVD molecule incorporates into the RNA primer strand and forms a base-stacking interaction with the guanosine. It also participates in strong hydrogen bonds with the uracil moiety from the RNA template strand. Thus, this structure actually represents a slightly different binding mode than FVP-RTP in 7CTT [81].

**Figure 9:**
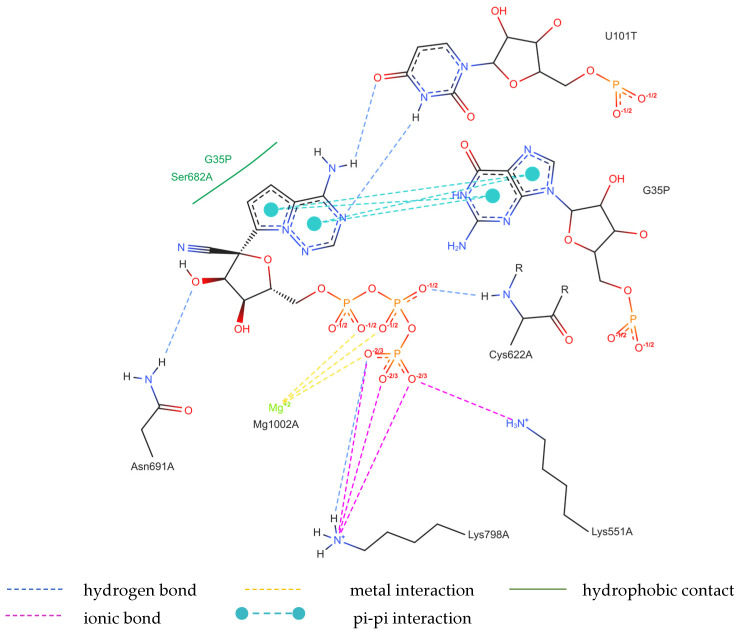
The binding mode of RDV (7UO4 [80]).

Considering the possibility of alternative binding of FVP-RTP to the RNA strand, we used 7UO4 [80] to simulate this particular variant. However, the actual ligand, RVD, binds to a similar set of the RdRp residues (ASP623, ASN691, ARG555, LYS798, LYS621, ARG553, LYS551 ASP618, ASP760, and SER682) and one magnesium ion (cofactor), as shown in Table 13 and Table 14 and Figure 9.

The binding of RVD to the hydrophilic cluster residues LYS545, ARG553, ARG555, and LYS551 and the Mg^2+^ cofactor is very strong and involves π-cation (ARG555), salt bridge (LYS551), or electrostatic/charge–charge (ARG553 and Mg^2+^) interactions. The interaction between RVD and LYS798 is mixed in nature and can be described as a combination of salt bridges and electrostatic (charge–charge, attractive) interactions. RVD interactions with ASP618, ASP760, and ASP761 are primarily electrostatic but repulsive. Ligand bonds with guanosine from the RNA primer and SER682 are of the π⋯π type (hydrophobic). Conventional strong and attractive hydrogen bonds bind RDV to ASP623, ASN691, and uracil. In-depth analysis reveals that the actual ligand, RVD, is linked to the uracil in the RNA template via two hydrogen bonds: N–H⋯O of 2.878 Å and N–H⋯N of 3.10 Å, involving the amine group and nitrogen N(4), respectively. In addition, three N–H⋯O hydrogen bonds link –OH moieties from RDV to ASN691 (of 2.80 Å), LYS798 (of 2.89 Å), CYS622 (of 3.44 Å), and two O–H⋯O hydrogen bonds link RDV with ASP623 (of 3.21 Å). The atoms of the RDV heterocyclic ring and nitrogen atom from the –NH_2_ group participate in the π⋯π stacking with guanosine (of 3.99–5.0 Å). The RVD ligand binds to the NH_3_^+^ group of LYS798 using three oxygen atoms of its PO_3_ moiety.

##### Binding Mode of the Candidate Ligands to RdRp

Docking Results

**Figure 10:**
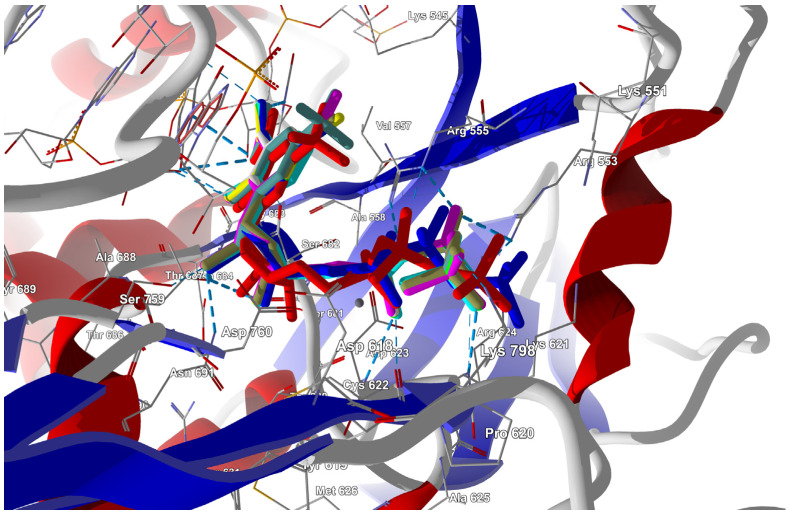
The docked poses of the ligands in the binding site of RdRp (target from 7UO4 [80]). The protein backbone is represented as a cartoon, the binding cavity residues are shown as thin sticks, and the docked ligands are shown as sticks.

The triphosphorylated forms of the candidate ligands in which the F at the C(6) position was replaced by I, Br, Cl, H, CH_3_, CF_3_, or CN were docked to the active site instead of the parental RDV. The docking results are summarized in Table 15, and the best poses are shown in Figure 10.

As shown in Table 15, the total protein–ligand binding energy increased by approximately 30% compared to the energy of the actual ligand, RDV. The highest docking score and total protein–ligand binding energy are observed for FVP-RTP and the CF_3_ and CN analogues are shown in Table 16. The ligands’ ordering according to the decreasing docking score is as follows:

F > CF_3_ > CN > Cl > Br > I > CH_3_ > H

According to the decreasing total energy of binding, the ligands can be ordered as follows:

CF_3_ > F > CN > CH_3_ > Cl > I > H > Br

The sum of the energies of binding ligand to cofactor, RNA Template, and RNA primer is the highest for the CH_3_ analogue, followed by F and CN. The protein–ligand hydrogen bonds are the strongest for FVP-RTP, followed by the H and Cl analogues.

In-Depth Analysis of the Binding Mode

Overall, the original FVP-RTP ligand, as well as its analogues, bind to the same set of RdRp residues, RNA primer, RNA template, and one cofactor (Mg^2+^ ion) but via different non-covalent interactions. In-depth analysis shows that the bindings of RVD and FVP-RTP to RdRp differ by as much as 20 residues (the Tanimoto distance 0.73). Differences in binding modes of specific ligands compared to the actual ligand RVD (the entire complex, only protein residues, only cofactors, RNA template, and RNA primer) calculated using different metrics (Euclidean or Manhattan) and additively are listed in Table 17 and Table 18.

In terms of overall interaction pattern, the CF_3_ analogue is closest to RDV, while the H and I analogues are the most different. However, the differences are mainly due to binding to the co-factors, RNA primer, and RNA template. The three analogues, F, CN, and CF_3,_ appear to be the most preferred because they exhibit the strongest total protein–ligand binding, as well as a strong binding ability to both the RNA primer and RN template. The differences between the interactions within the entire complex and active site residues, as shown in Table 17 and Table 18, are subtle, so only residues near the ligand play a key role. Although each ligand interacts with as many as 83 residues of RdRp, most of them are of minor importance, Figure S3.

Docked ligands interact mainly with the active site residues via hydrogen bonding and hydrophobic interactions. Detailed insight into the binding mode using Ligplot+ [82,83] confirms the presence of the hydrogen bonds and reveals the hydrophobic interactions binding the ligands and ASP760, THR687, ASP623, VAL557, ASP618, adenosine, and uracil, as shown in Figure S4. Note that Ligplot+ suggests a similar set of hydrophobic interactions for the ligands binding to the same protein. However, only FVP-RTP forms an NH⋯O hydrogen bond with ASP623 and ASP760. In the case of the CF_3_ analogue, the hydrophobic contacts replace the hydrogen bonds. The CF_3_ analogue has a high docking score but relatively low binding affinity, so it may seem most convenient when it is desired to exclude binding to uracil.

**Figure 11:**
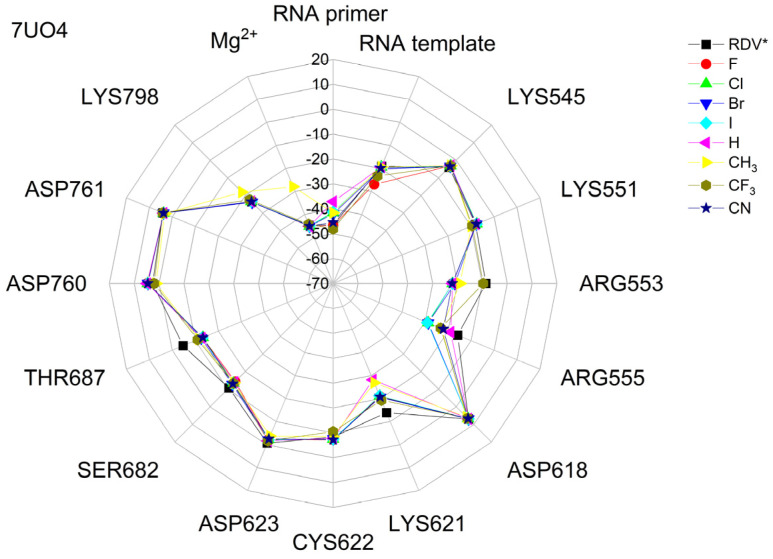
The comparison of the binding energies of FVR-RTP analogues with RdRp (selected residues), RNA primer, RNA template, and cofactor (target from 7UO4 [80]). (RDV*—native ligand).

The binding energies of FVR-RTP analogues with the RdRp residues, RNA primer, RNA template, and cofactor are summarized in Table 19, and a radar plot comparing these data is shown in Figure 11.

As follows from Table 19 and Figure 11, the replacement of the F at C(6) with I, Br, Cl, CF_3_, H, CH_3_, CF_3_, and CN leads primarily to the changes in the binding strength of the ligand to the RNA primer, Mg^2+^, RNA template, and residues: LYS545, LYS551, ARG553, ARG555, ASP618, LYS621, CYS622, ASP623, SER682, THR687, ASP760, ASP761, and LYS798. The nature of these interactions is the same as previously described for 7CTT [81], but their strengths are different, e.g., the bond with Mg^2+^ is very strong. Therefore, the distribution of the binding energy between individual residues is relatively uniform, Figure 11. Moreover, some residues, such as ARG553, ARG555, LYS621, and THR687, are highly sensitive to the type of the ligand. The high similarity of the binding modes in candidate ligands to RDV, greatest for the CF_3_ analogue, is confirmed by the high cosine distance values.

Binding Mode Visualization

**Figure 12:**
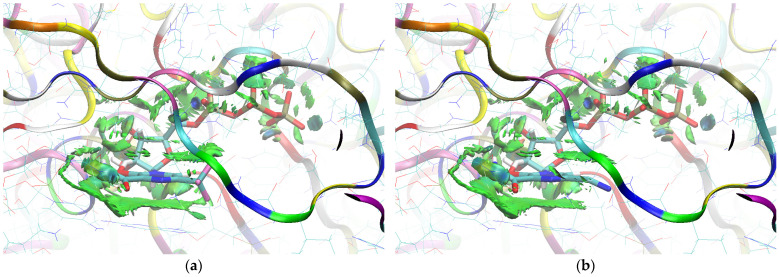
The isosurfaces of RDG (isovalue 0.5a.u.) with sign(λ_2_)ρ_BCP_ mapped over the surface for (**a**) the CF_3_ analogue and (**b**) the CN analogue.(Pre-Catalytic State—Productive Mode II).

The sign[λ_2_(r)]ρ(r) surface mapped to the RDG(r) isosurface in the red–green–blue scheme visualizes the binding mode for the RdRp-ligand complex and reveals the nature of the non-covalent interactions between RdRp and candidate ligands, shown in Figure 12.

Weak van der Waals-type interactions clearly dominate, as indicated by the green color of the surface. The N–H⋯O hydrogen bond linking the ligands to the RNA strand is clearly visible and depicted by a small light cyan disc-shaped region near =O and -NH_2_. The large and nearly flat green surfaces above the six-membered ring of the FVP ring prove π⋯π stacking between the ligands and guanosine. The differences between the modes of binding of the CF_3_ and CN analogues are small, as shown in Figure 12.

However, the differences between the binding modes in both pre-catalytic states (binding to cytosine and stacking to adenosine vs. binding to uracil and stacking to guanosine) for the same ligands are significant as the cosine distance ranges from 0.550 to 0.610. The most significant difference concerns the binding of ligand to Mg^2+^, which is six times stronger. Three residues, ARG555, ARG553, and ASP618, are important, but their ligand binding is only twice as strong. Ligand binding to the RNA template is only slightly stronger. The docking results suggest that among the analogues studied, the CF_3_ derivative should bind most strongly to RdRp, the cofactor Mg^2+^, the RNA template, and the RNA primer and may therefore be a very good alternative to FVP-RTP and RVD. The key factor for improving FVP effectiveness seems to be increasing the binding strength of the ligand to the RNA template.

#### 2.3.3. Pre-Catalytic State—Non-Productive Mode

The structure of the complex of the SARS-CoV-2 replicating polymerase bound to FVP-RTP in the pre-catalytic and non-productive state (7AAP) [84] was retrieved from the PDB database. The pocket containing FVP-RTP has a surface area of 1120.59 Å^2^, a volume of 1205.75 Å^3^, and therefore a surface/volume ratio of 0.93. Its hydrophobicity is 0.61. The 7AAP [84] backbone differs from 7UO4 [80] by 0.737 (backbone residues) and 1.638 (all residues), which is almost twice as much as 7CTT [81]. The FVP-RTP molecule in 7AAP [84] weakly interacts with ASP618 (5.238 kcal/mol), SER759 (−0.79 kcal/mol), ASP760 (1.779 kcal/mol), and ASP761 (6.171 kcal/mol) but binds strongly to LYS545 (−9.426 kcal/mol), ARG553 (−4.621 kcal/mol), and ARG555 (−8.070 kcal/mol). In the non-productive mode, the phosphoribosyl conformation is different than in the productive mode and ensures a very strong binding of FVP-RTP to two Mg^2+^ ions (−28.57 and −44.10 kcal/mol).

**Figure 13:**
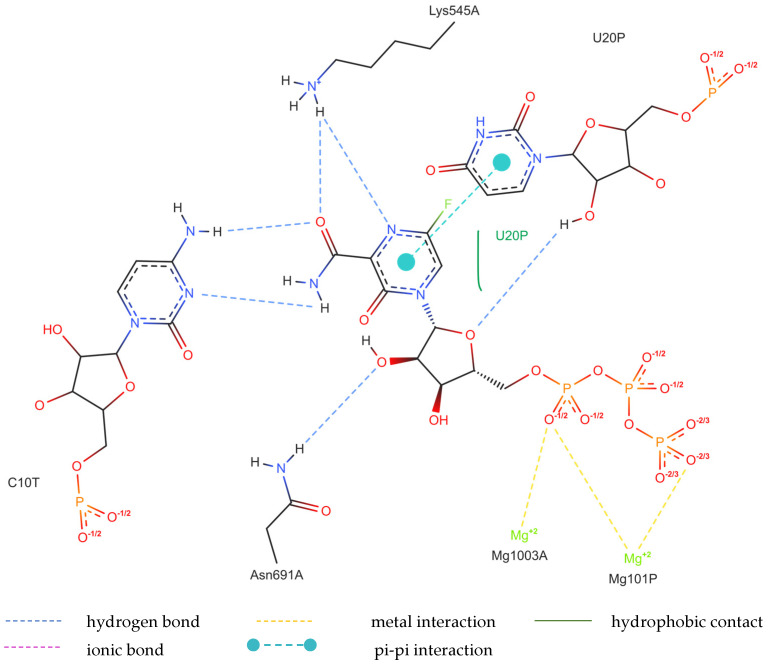
The binding mode of FVP-RTP in 7AAP [84].

FVP-RTP interactions with the hydrophilic cluster residues LYS545, ARG553, ARG555, and LYS551, as well as the Mg^2+^ cofactor, are very strong and include π-cation (ARG555), salt bridge (LYS551) or electrostatic/charge–charge interactions (ARG553, Mg^2+^). The bonding between FVP-RTP and LYS798 exhibits a mixed nature and can be described as a combination of salt bridges and electrostatic (charge–charge, attractive) interactions. The FVP-RTP interactions with ASP618, ASP760, and ASP761 are mainly electrostatic but repulsive. The interactions of the ligands with guanosine from RNA primer and SER682 are of the π⋯π type (hydrophobic). Conventional strong and attractive hydrogen bonds bind RDV to ASP623, ASN691, and uracil. In-depth analysis reveals that the actual ligand, FVP-RTP, is linked to uracil in the RNA template via two hydrogen bonds, N–H⋯O of 2.878 Å and N–H⋯N of 3.10 Å, involving the amine group and nitrogen N(4), respectively, Figure 13.

##### Docking Results

The docking results of FVP-RTP and its seven analogues are summarized in Table 20. Negative values of the docking scores suggest generally favorable docking of the ligands at the binding site.

As shown in Table 20, the docking score values are the highest for the CF_3_ analogue, followed by CN and I.

The ordering of the ligands in descending order of the docking scores is as follows:

CF_3_ > CN > I > Br > F > H > Cl > CH_3_

According to the decreasing total energy of binding, the ligands can be ordered as follows:

CF_3_ > Br > Cl > I > CH_3_ > CN > H > F

The total protein–ligand binding energy for the CF_3_ analogue was increased by 20% compared to that of the actual ligand, FVR-RTP. The sum of the binding energies of the ligand to the cofactor, RNA Template, and RNA primer is highest for the CH_3_ analogue, followed by CF_3_ and Br (this order is mainly due to the steric effect). Hydrogen bonds between protein and ligand are strongest for the CF_3_ analogue, followed by CH_3_ and H. The CF_3_ analogue’s highest binding affinity suggests it is the most promising ligand. On the contrary, the lowest binding affinity of the CN analogue suggests that it may offer a non-productive mode exclusion.

##### In-Depth Analysis of the Binding Mode

Differences in the binding modes of specific ligands compared to the actual ligand, RVD, calculated using different metrics (Euclidean or Manhattan) and additively are summarized in Table 21 and Table 22. Calculations were performed for three variants: the entire complex; protein residues; cofactors, RNA template and RNA primer.

The binding of the cofactors, RNA primer, and RNA template is the key factor that determines changes in the entire complex. The distinctions among the interactions throughout the entire complex and the active site residues are subtle, validating that only specific residues in close proximity to the ligand are crucial. In terms of the total interaction pattern, the CH_3_ analogue is the closest to the actual ligand FVR-RTP, while the CF_3_ and I analogues are the most different, as can be seen in Table 21 and Table 22. However, the differences in the strength of the residues’ interactions with individual ligands in 7AAP [84] are significantly greater than those observed in 7CTT [81] (especially for CF_3_ and I analogues). Overall, of the analogues tested, the CF_3_ derivative should bind most strongly to both RdRp and the cofactor, RNA template, and RNA primer, while the CN analogue provided the weakest binding.

The docked ligands interact mainly with the active site residues via hydrogen bonding and hydrophobic interactions. A thorough examination of binding modes using Ligplot+ uncovers the hydrophobic interactions that bind the ligands with ASP760, THR687, SER682, adenosine, and uracil, as shown in Figure S5.

The CF_3_ analogue additionally interacts with Asp618, while CN interacts with Asp555 and Asp623. Moreover, the CF_3_ derivative forms NH⋯O, NH⋯N, and NH⋯F hydrogen bonds with LYS545, while the CN analogue forms only one NH⋯N hydrogen bond. These bonds are absent in the structure of the 7AAP [84] complex. Thus, the binding mode in non-productive 7AAP is heavily influenced by the substituent.

**Figure 14:**
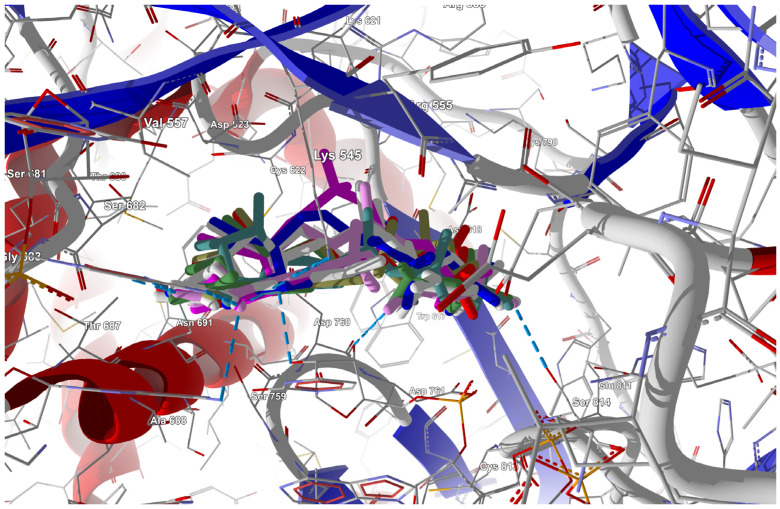
The docked poses of the ligands in the binding site of RdRp (target from 7AAP [84]). The protein backbone is represented as a cartoon; the binding cavity residues are shown as thin sticks, and the docked ligands are shown as sticks (FVP-RTP in cyan, the CF3 analogue in pink, and the CN analogue in white).

The binding energies of the FVR-RTP analogues to the RdRp residues, RNA primer, RNA template, and cofactor are summarized in Table 23, and the best poses are shown in Figure 14.

**Figure 15:**
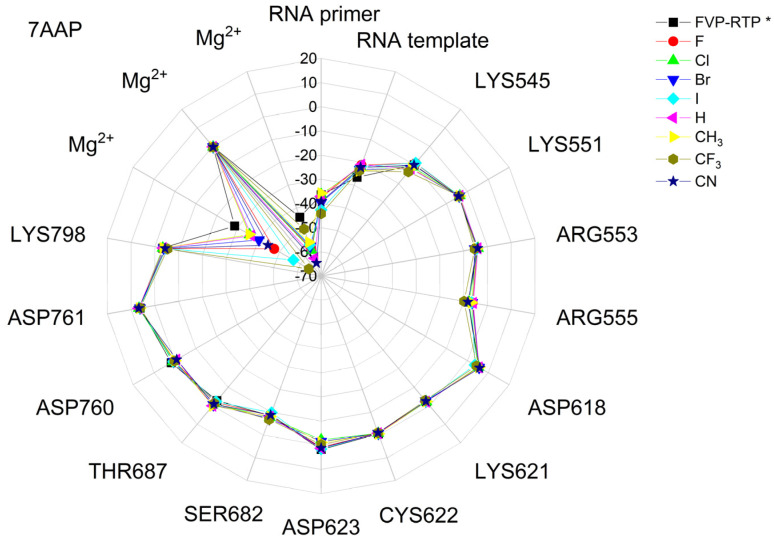
The comparison of the binding energies of FVR-RTP analogues to RdRp (selected residues), RNA primer, RNA template, and cofactor (target from 7AAP [84]) (FVP-RTP*—native ligand).

A radar plot illustrating the discrepancies in binding modes is shown in Figure 15.

The largest variations in binding strength caused by the substituent effect are observed in the bonds connecting the ligand and the cofactor. Most residues, except LYS545 and ARG555, are insensitive to the type of ligand. The strong resemblance of binding modes in the complexes of RdRp with candidate ligands and the complex with FVP-RTP is reaffirmed by the elevated cosine distance values. The highest cosine distance value for the CF_3_ analogue revealed its high similarity to FVP-RTP.

##### Binding Mode Visualization

**Figure 16:**
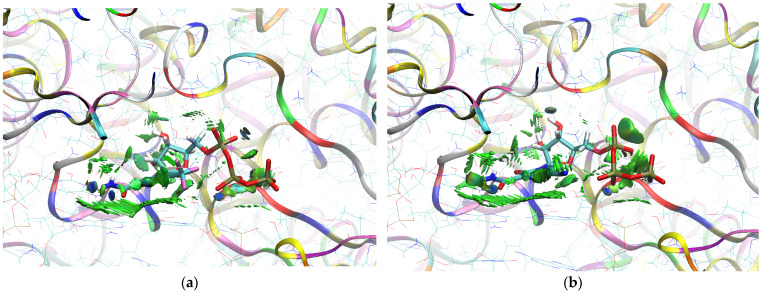
The isosurfaces of RDG (isovalue 0.5a.u.) with sign(λ_2_)ρ_BCP_ mapped over the surface for (**a**) the CF_3_ derivative and (**b**) the CN derivative (Pre-Catalytic State—Non-Productive Mode).

The sign[λ_2_(r)]ρ(r) surface mapped to the RDG(r) isosurface in the red–green–blue scheme for RdRp-ligand complex visualizes the binding mode and reveals the nature of the non-covalent interactions between the RdRp and the candidate ligands, as shown in Figure 16.

There are extensive green areas indicating the presence of weak van der Waals interactions, especially in the case of the CN derivative. The N–H⋯O hydrogen bond linking the ligands to cytosine is clearly visible and depicted as a small light cyan disc-shaped area near =O and -NH_2_. Both its shape and color reveal that the CF_3_ analogue binds more strongly to cytosine than the CN analogue. The large and nearly flat green area above the six-membered ring of the pyridazine ring proves π⋯π stacking between the ligands and guanosine. The CN analogue shows larger surfaces representing the interactions of phosphate groups with RdRp. However, the mostly green color indicates that these interactions are weaker than those observed for CF_3_.

The binding modes in 7AAP [84] and 7OU4 [80] differ in the binding strength within the complex components, i.e., the RNA primer, LYS621, LYS798, and Mg^2+^. The most significant differences between the non-productive (7AAP [84]) and productive modes (7CTT [81] and 7OU4 [80]) concern the binding strength of LYS798 and Mg^2+^ (three magnesium ions involved) with the ligand, which are six times weaker and very strong, respectively. The inorganic Mg^2+^ cofactor, which binds strongly to each ligand in the non-productive mode, changes the structure and chemical potential of the active site. The LYS798 residue, which stabilizes the core of the RdRp, is weakly bound by the ligand in this mode. The CF_3_ analogue shows the strongest binding to LYS798 among the FVP-RTP analogues and forms a strong binding to the cofactor. Moreover, the binding of the ligand to the RNA primer in the productive state is weaker, but to the RNA template, it is stronger. Importantly, the binding affinity in the non-productive mode is not correlated with the R+ and R-values.

The differences between the binding modes in productive and non-productive states can be compared using the radar plots in Figure 7, Figure 12 and Figure 15. In the productive modes, the bindings between the ligands and LYS621, LYS798, ARG555, and ARG553 are stronger than in the non-productive mode. Furthermore, the allocation of interaction energy between the active-site residues is more evenly distributed in the non-productive mode (i.e., the interactions are less directional).

#### 2.3.4. Active State

The development of nucleotide-based medicines for combating COVID-19 relies on the understanding of the structure in the active state. The structure of the replicating polymerase complex of SARS-CoV-2 in the active state bound to FVP-RMP (7DFG) [85] was retrieved from the PDB database. The pocket containing FVP-RMP incorporated into the RNA strand has a volume of 2765 Å^3^. The two most promising ligands (the CF_3_ or CN analogues of FVP-RMP) were prepared, incorporated into the RNA strand, and docked.

##### Docking Results

**Figure 17:**
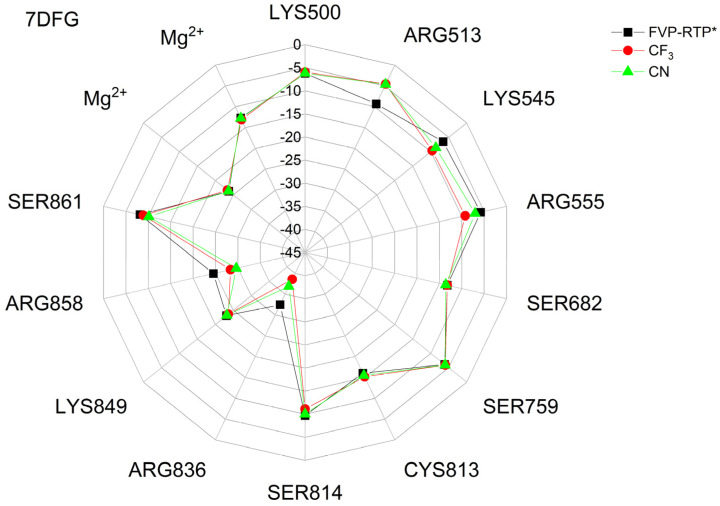
The comparison of the binding energies of the FVR-RMP analogues to RdRp (selected residues) and cofactor (target from 7DFG [85]). (FVP-RMP*—native ligand).

The docking results are summarized in Table 24, and a radial plot comparing these data is shown in Figure 17.

##### In-Depth Analysis of the Binding Mode

The docking score is higher for the CN analogue than for the CF_3_ analogue, but the total energy of protein–ligand binding and hydrogen bond strength are higher for CF_3_ than for CN, as shown in Table 25.

**Figure 18:**
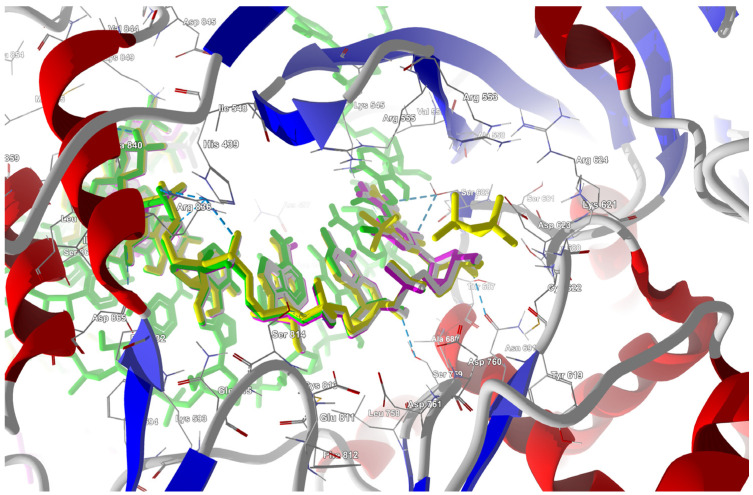
The docked poses of the ligands in the binding site of RdRp (Active state). The protein backbone is represented as a cartoon; the binding cavity residues are shown as thin sticks and docked ligands are shown as sticks (FVP in pink, the CF_3_ analogue in yellow, and the CN analogue in green).

Furthermore, the CF_3_ analogue has a much higher (−75.135 kcal/mol), while the CN analogue has a lower (−69.515 kcal/mol) binding affinity than the actual ligand (−72.457 kJ/mol). The radial plot presented in Figure 17 demonstrates uniformity and symmetry similar to that observed in the pre-catalytic state (target from 7CTT [81]) that was previously described. Substitution leads to significant differences in binding strengths of ARG836, ARG858, ARG513, ARG555, and LYS545, with ARG836 being most strongly affected. However, determining which ligand has a binding mode closest to FVP-RMP from examining just the plot shapes poses a challenge. In this task, the cosine distance is helpful. The cosine distance between the energetic profiles of binding of both ligands is high (0.996), which confirms their high similarity and binding efficiency. Moreover, the cosine distance between the energetic profiles of binding for FVP-RMP and the CN analogue incorporated in the RNA strand is slightly higher than that of FVP-RMP and the CF_3_ analogue (0.993 vs. 0.991, respectively). Thus, in terms of the overall interaction pattern, the CN analogue is closer to FVP-RMP than the CF_3_ analogue. The distinctions between the two ligands primarily stem from their bindings to the co-factors and RNA template. There is relatively little variation in the strength of the bindings within the active site residues. The best poses with the greatest binding/docking scores, illustrated in Figure 18, appear to be insignificantly distinct.

The disparities in the binding modes of particular ligands compared to the actual ligand, ascertained using various metrics (Euclidean and Manhattan) and additively, are summarized in Table 26 and Table 27. Three variants, the entire complex, protein residues, and cofactors and the RNA template, were taken into account.

Detailed insight into the structures of protein–ligand complexes using Ligplot+ reveals the hydrophobic interactions between the candidate ligands incorporated into the RNA strand and RdRp residues, Figure S6. As can be seen from Figure S6, all ligands interact with the residues ASP623, CYS813, ALA840, SER861, and MET855. Additionally, FVP-RTP interacts hydrophobically with ASP760 and ASP865; the CF_3_ analogue interacts with ASP691, LYS849, and GLU857; and CN interacts with LEU862, LYS849, and GLU857. Furthermore, both CN and CF_3_ analogues form OH⋯O hydrogen bonds with ASP865, while ASP760 forms metallic bonds with Mg^2+^ instead of the ligand. These bonds are not present in the current active state complex. Thus, the nature of the binding mode in the active state is strongly modulated by the substituent. Additionally, the hydrogen bond NH⋯F of 3.299Å between Lys545 and the CF_3_ ligand results in the RNA strand with the CF_3_ analogue being held more securely in the hydrophilic pocket. The CN analogue does not form an extra bond. Nevertheless, thanks to the -CN group, it facilitates water binding.

##### Binding Mode Visualization

**Figure 19:**
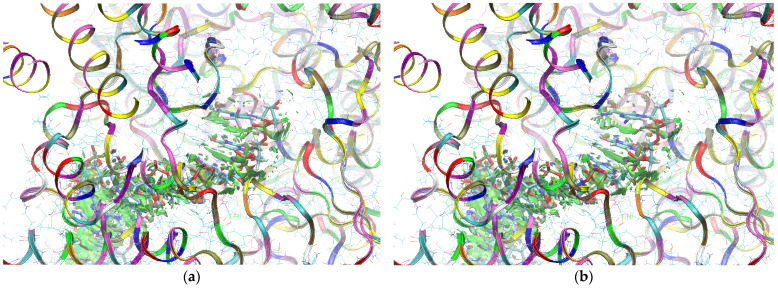
The isosurfaces of RDG (isovalue 0.5a.u.) with sign(λ_2_)ρ_BCP_ mapped over the surface for (**a**) the CF_3_ derivative and (**b**) the CN derivative. (Active state).

The mapping of sign[λ_2_(r)]ρ(r) onto the RDG(r) isosurface demonstrates the significant similarity between the binding modes of the CF_3_ and CN analogues, as shown in Figure 19.

Green areas in Figure 19 indicate weak non-covalent interactions. The green, flat, and almost parallel surfaces in the single-stranded RNA template reveal numerous instances of hydrophobic π⋯π stacking between aromatic rings.

#### 2.3.5. Allosteric Effect

A possible alternative mechanism of FVP action, which may explain the scattering of the results of clinical trials [86,87] or the synergistic effect observed in combined treatment against SARS-CoV-2 [88], can be associated with the allosteric effect [64]. The cryo-electron microscopy structure of SARS-CoV-2 virus full-length nsp12 in complex with cofactors nsp7 and nsp8 (6M71) [74] was retrieved from the PDB database. The allosteric sites were detected with Allosite-Pro [89] and PASSer [90] using a scheme [64,91,92].

Near the active site (2943 Å^3^ in volume), there are two allosteric pockets on either side of the RNA strand. One of them is composed of VAL557, ARG555, LYS545, THR680, SER682, THR556, ASP623, THR687, SER759, ASN691, ALA688, ASP760, and ARG553.

##### Docking Results

**Figure 20:**
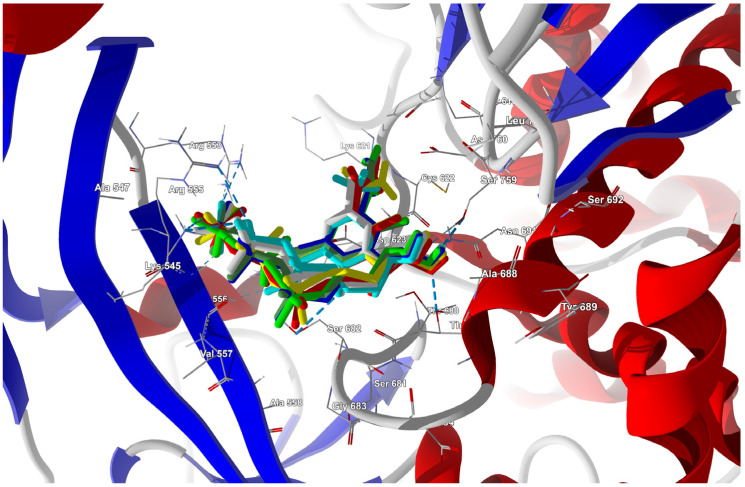
The docked poses of the ligands in the binding site of RdRp (the RdRp target from 6M71 [74]). The protein backbone is represented as a cartoon; the binding cavity residues are shown as thin sticks and docked ligands are shown as sticks (FVP in pink, the CF_3_ analogue in yellow, and the CN analogue in green).

Ligands in which the F at the C(6) position was replaced by I, Br, Cl, H, CH_3_, CF_3,_ or CN were docked to this site. The docking results are summarized in Table 28, and the best poses are shown in Figure 20.

The ligands’ ordering according to the decreasing docking score is as follows:

F > I > CN > Br > CF_3_ > CH_3_ > Cl > H

According to the decreasing total energy of binding, the ligands can be ordered as follows:

F > I > CN > Br > CF_3_ > H > CH_3_ > Cl

The ligands’ ordering according to the decreasing binding affinity is as follows:

CF_3_ > Br > I > F > Cl > H > CN > CH_3_

The CF_3_ analogue binds directly to the RdRp, much weaker than the F or CN ones. This direct binding of the CF_3_ analogue is also much weaker than when it is incorporated into the RNA strand.

##### In-Depth Analysis of the Binding Mode

Comparison of the cosine distance between the binding energy profiles of the potential ligands indicates that the CF_3_ analogue deviates most from others in terms of overall interaction pattern.

The conformation of FVP-RTP docked directly into the allosteric pocket is very similar to its conformation in the RNA template, Figure 18.

**Figure 21:**
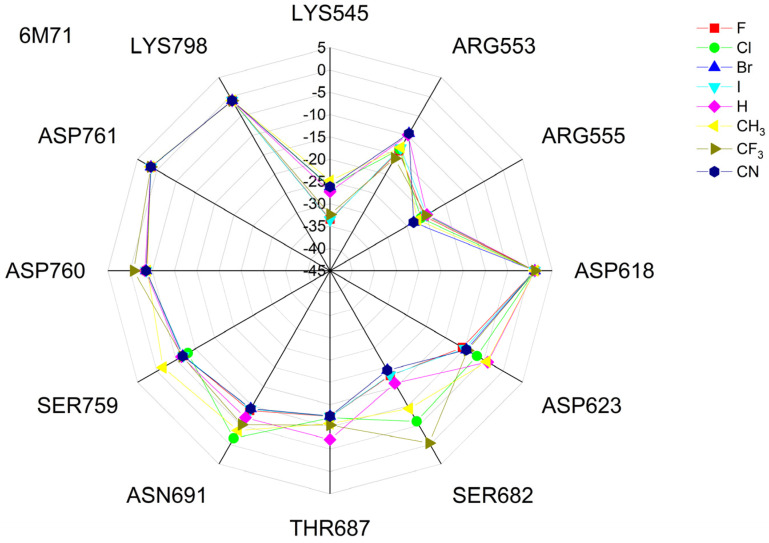
The comparison of the binding energies of the FVR-RTP analogues to RdRp (the RdRp target from 6M71 [74]).

Docking of the FVP-RTP analogues directly into the allosteric pocket leads to their very strong bindings to hydrophilic cluster residues LYS545, ARG555, and ARG553, but much weaker to conserved residues ASP623 and ASP618 and critical catalytic residues SER682, SER759, ASP760 and ASP761, as shown in Table 29 and Figure 21.

Changing the ligand has no effect on binding to LYS798 and ASP761, while bindings to LYS545, SER682, and ASN691 are strongly modulated by the ligand, as shown in Figure 21. Surprisingly, despite the high binding affinity for the CF_3_ derivative, the cosine distance to the F analogue is the largest, which is mainly due to the weak binding to SER682.

Detailed insight into the structures of protein–ligand complexes using Ligplot+ reveals the hydrophobic interactions between the candidate ligands incorporated into the RNA strand and RdRp residues, Figure S7. As can be seen from Figure S7, all ligands interact hydrophobically with the residues ARG555, VAL557, and ALA688. Additionally, FVP-RTP interacts hydrophobically with CYS622, ASP623, ASP691, and ASP760, while the CN analogue interacts with SER682, CYS622, and THR556. Furthermore, FVP-RTP forms OH⋯O hydrogen bonds with SER759 and NH⋯O with ASN691, ASP623, TYR556, ARG553, and LYS545. The CF_3_ analogue forms OH⋯O hydrogen bonds with SER759 and THR676, and NH⋯O bonds with Arg553 and LYS545, while the CN analogue forms OH⋯O bonds with SER759 and NH⋯O bonds with ASP760, ASP623, THR687, ARG555, and LYS545. Thus, their binding modes differ significantly.

##### Binding Mode Visualization

**Figure 22:**
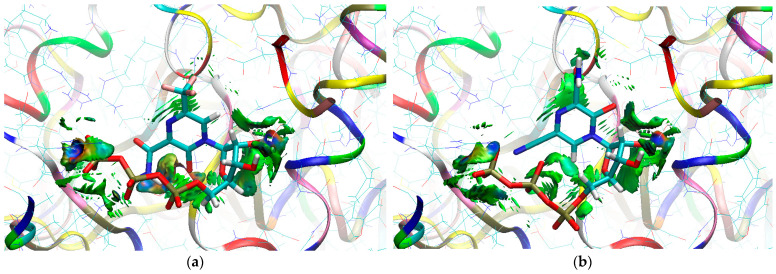
The isosurfaces of RDG (isovalue 0.5a.u.) with sign(λ_2_)ρ_BCP_ mapped over the surface for (**a**) the CF_3_ derivative and (**b**) the CN derivative. (Allosteric effect).

The mapping of sign[λ_2_(r)]ρ(r) onto the RDG(r) isosurface in Figure 22 demonstrates above-mentioned differences between the binding modes of the CF_3_ and CN analogues.

Green areas in Figure 22 indicate non-covalent interactions. The green and flat regions reveal hydrophobic π⋯π stacking, while cyan discs indicate hydrogen bonds.

### 2.4. Comparison of the Productive, Non-Productive, Active State and Allosteric Site Binding Modes

Upon comparison of all protein–ligand complexes described above, it is apparent that the main channel proteins (LYS545, ARG553, and ARG555) are relatively loosely bound to the ligand in the non-productive and active states. Nevertheless, in the pre-catalytic states and in instances of direct binding, the binding is significantly stronger. The ligand binding to LYS798 firmly stabilizes the RdRp core in pre-catalytic states, but the binding is feeble in non-productive and direct binding states.

**Figure 23:**
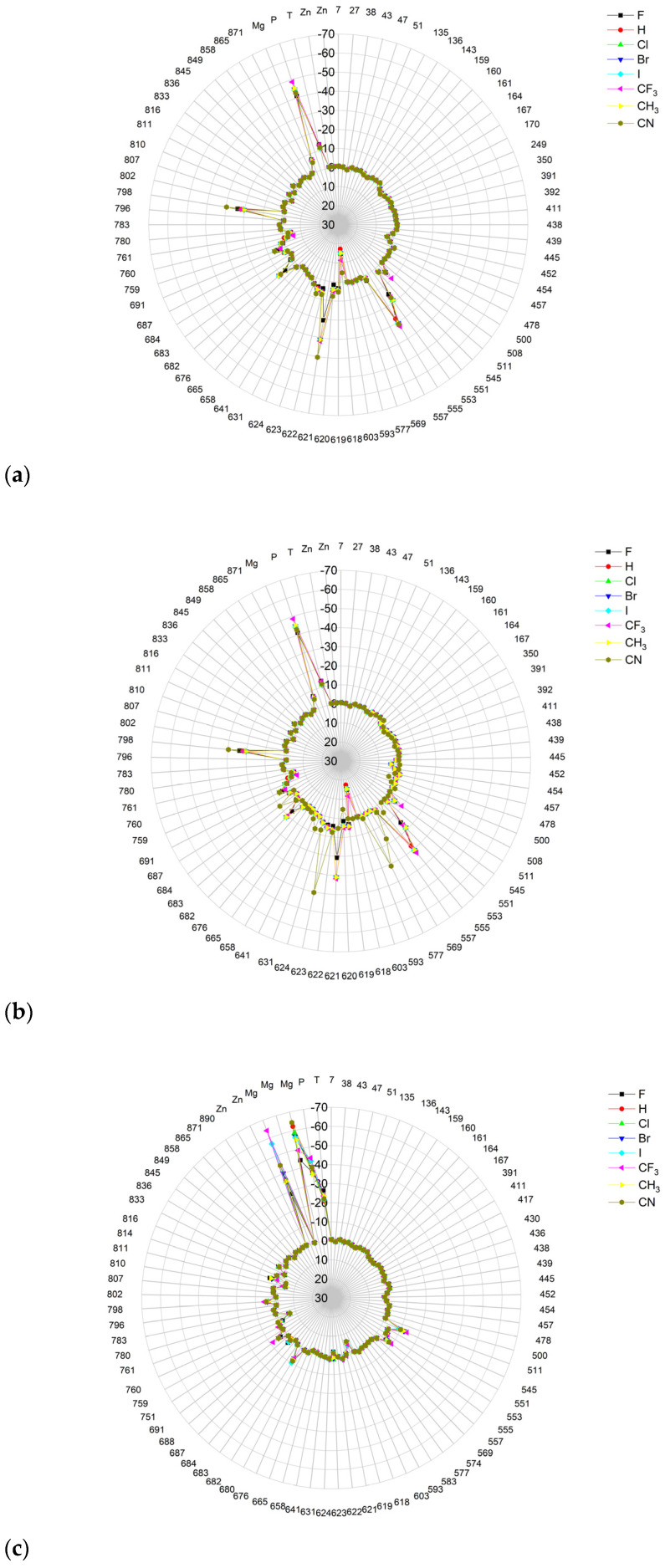
The comparison of the binding energies of the FVR-RTP analogues to RdRp of all residues, cofactors Mg^2+^ and Zn^2+^, RNA Template, and RNA Primer; (**a**) pre-catalytic productive mode I; (**b**) pre-catalytic productive mode II; (**c**) non-productive mode. Pop-up peaks represent components with high binding energy.

Radar plots, as shown in Figure 23, aid in quick screening, and highly bonded components are easily identified with pop-up peaks.

The CN analogue exhibits lower selectivity, as it promotes comparable binding modes in pre-catalytic state I and II. Conversely, the CF_3_ analogue primarily reinforces pre-catalytic state I. The latter, combined with a better ADME profile, increases CF_3_ attractiveness in terms of selective anti-virial activity. Strong direct binding of FVP-RTP to the active site residues suggests a possible alternative mechanism of FVP action, which may explain the scattering of the results of clinical trials [25,63] or the synergistic effect observed in combined treatment against SARS-CoV-2 [64]. The replacement of F with CF_3_ may help to overcome this limitation.

## 3. Methods

### 3.1. ADME and Drug-Likeness Prediction

In silico ADME drug-likeness evaluation was performed using the SwissADME tool developed by the Swiss Institute of Bioinformatics [66]. Drug-likeness was tested according to Lipinski, Veber, and Egan’s rules of 5 (RO5). The Abbot Bioavailability scores were computed to predict the probability of a compound having at least 10% oral bioavailability. Lipophilicity was predicted with iLOGP, XLOGP3, WLOGP, MLOGP, and SILICOS-IT models, from which a consensus log Po/w was determined [66]. The solubility (log S) of the ligands was predicted using SILICOS-IT [66]. The mutagenicity and carcinogenicity were predicted using models implemented in ADMET2.0 [71]. Synthetic accessibility (SA) was predicted based on the assumption that the frequency of molecular fragments in “really” obtainable molecules correlates with the ease of synthesis [66].

### 3.2. Density Functional Theory

Quantum chemical calculations were carried out within the Density Functional Theory (DFT) approach rooted in the Kohn–Sham [93] theorem, generalized by Levy [94]. Becke’s hybrid exchange-correlation functional, B3LYP [95,96]; Møller-Plesset, MP2 [97]; Minnesota hybrid meta M062X a high-nonlocality exchange-correlation functional with double the amount of nonlocal exchange (2X) [98], and the all-electron split-valence basis set 6-311G(d,p) were used to optimize the geometry of the ligands and their triphosphate forms. Geometry optimization was performed in the internal coordinates system. Following geometry optimization, a Hessian matrix (a matrix of second derivatives of the energy with respect to atomic coordinates) was calculated for all optimized ligand structures to verify the absence of imaginary frequencies (i.e., true stationary points). The polarizable continuum model (PCM) [99] was used to model solvation effects, required for the estimation of the reactivity indices in solution. The calculations were performed using Gaussian 16 rev. C01 [100].

The theoretical reactivity indices defined by Par and Pearson [101], Gazquez [102], and Chattaraj [103] are as follows: the absolute electronegativity [χ = −(E_LUMO_ + E_HOMO_)/2; eV]; absolute hardness [η = (E_LUMO_ − E_HOMO_)/2; eV)]; electrophilicity index (reactivity) [ω = χ^2^/2η; eV]; softness [S = 1/η; 1/eV]; electro-donating power [ω^−^; eV]; electro-accepting power [ω^+^; eV]; net electrophilicity [Δω = ω^+^ + ω^−^; eV]; and maximum number of electrons transferred in a chemical reaction [ΔN_max_]. These indices were evaluated at MP2/6-311G(d,p) and M062X/6-311G(d,p), and describe the effect of donating (HOMO) and accepting (LUMO) electrons in the frozen core approximation (the Koopmans theorem [104]).

### 3.3. Quantum Theory of Atoms in Molecules

Theoretical analysis of the topology of intermolecular interactions was performed within Bader’s Quantum Theory of Atoms in Molecules (QTAIM) [105] approach using Gaussian 16 rev. C01 [100]. The DFT wavefunction was calculated at the M062X/6-311+G(d,p) level of the theory. The so-called topological descriptors calculated at the Bond Critical Points (BCPs) include the electron density at BCP, ρ(r_BCP_); three eigenvalues of the principal components of Hessian matrix composed of second partial derivatives of the electron density describing curvature of the electron density according to the principal axes λ_1_, λ_2_, and λ_3_; and Laplacian (the sum of Hessian eigenvalues), Δρ. Some of these descriptors (λ_2_ and Δρ) provide information about the nature and strength of the interactions. In many molecular systems, the reduced density gradient (RDG), which is highly sensitive to the electron density distribution and its variations, helps to detect weak or specific non-covalent interactions that were uncertain or not revealed by classical QTAIM [105,106].

The reduced density gradient is defined as follows:

(3) $$RDG(r)=\frac{\left|\nabla \rho (r)\right|}{{2(3\pi )}^{1/3}{\rho (r)}^{4/3}}$$

where ∇ρ(r) is the electron density gradient, and ρ(r) is electron density. The RDG(r) vs. sign(λ_2_)ρ(r) plot reveals characteristic spikes in the low-gradient and low-density regions as long as the non-covalent contacts are present in the structure. The nature of these contacts can be determined using the sign of λ_2_, which allows their classification as attractive (stabilizing; λ_2_ < 0) or repulsive (destabilizing; λ_2_ > 0). A spike in the low-gradient, low-density region at λ_2_ < 0 suggests a stabilizing interaction such as a hydrogen bonding, a smaller spike accompanied with only slightly negative λ_2_ indicates a weakly stabilizing interaction, and a spike associated with λ_2_ > 0 indicates the absence of non-covalent interactions. Thus, the reduced density gradient (RDG) isosurface analysis allows for the analysis and visualization of non-covalent repulsive and attractive interactions. The results of the RDG analysis were visualized using Visual Molecular Dynamics (VMDs) [107]. The isosurfaces have been colored using a blue–green–red scale, where blue indicates strong attraction, red indicates strong repulsive interactions, and green indicates weak van der Waals interactions.

### 3.4. Molecular Docking

Molecular docking is a widely used technique in drug development for the screening of novel therapeutic agents. It requires knowledge of the reliable 3D structures of the ligand and target. The structures of the candidate ligands were optimized at the M062X/6-311+G(d,p) level, while the target RdRp structures were downloaded from the Protein databank PDB database (http://www.rcsb.org/pdb, accessed on 30 September 2023). The structures of the replicating polymerase complex of SARS-CoV-2 with FVP-RTP (7CTT) [81], RVD (7UO4) [80], FVP-RMP(7AAP) [84], and FVP-RTP (7DFG) [85] were retrieved from the PDB database. Prior to docking the candidate ligands, the native ligand that co-crystallized with the RNA was removed from the structure. The docking was performed using candidate ligands incorporated into the RNA strand. The cryo-electron microscopy structure of SARS-CoV-2 full-length nsp12 in complex with cofactors nsp7 and nsp8 (6M71) [74] was used exclusively for the studies of allosteric effect. The allosteric sites were detected with software dedicated for this purpose, i.e., Allosite-Pro [89] and PASSer [90], using schemes [64,91,92]. Allosite-Pro 2016 predicts allosteric sites using Support Vector Machines (SVMs), while PASSer 2021 uses eXtreme gradient boosting (XGBoost) and graph convolutional neural networks (GCNNs). However, both methods are based on a set of topological and physicochemical parameters. The results obtained from both methods were very similar. The active site differs only slightly in shape and size but not in position.

Conversion of the files with receptor and ligand structures to the .pdbqt format was carried out using MGLTools ver. 1.5.7. Molecular docking was performed using the automated docking tools AutoDock ver. 4.2.6 [108] and AutoDock Vina ver. 1.2.3 [109]. Both are designed to predict the binding of small molecules, such as substrates or drug candidates, to a receptor. AutoDock calculations were performed in three steps: (1) the preparation of coordinate files using AutoDockTools, (2) the pre-calculation of atomic affinities using AutoGrid, and (3) the docking of ligands using AutoDock. AutoDock AutoGrid was used to pre-calculate the grids for each type of atom in the ligand, as well as grids of electrostatic and desolvation potentials. AutoDock Vina automatically performs this task. The pre-calculated grid was treated as a template. Two techniques were used for docking: Template docking based on the native ligand and docking within a defined searched space around the active site. The search space was a grid box with step sizes covering 9–15 Å centered on the active site. The Autodock was applied for the docking of the ligand to a set of grids describing the target protein. Some specific sidechains (in the nearest neighborhood of the cavity) were treated as flexible. For docking, we applied the most computationally efficient method: a Lamarckian genetic algorithm (LGA) with only a slightly modified set of parameters.

To evaluate the quality of the docking process, we performed a redocking task. The actual ligand was removed from the parental structure and redocked in its own binding site. The redocking protocol was considered successful when the root-mean-square deviation (RMSD) of pose relative to its conformation in the parental structure did not exceed 3 Å. Then, in the same way, the new ligand was docked into the rigid protein structure. The number of rotatable bonds in the ligands was calculated, and the ligands were treated as flexible bodies. Due to the high flexibility of the ligands, as many as 50 conformational poses were searched for each ligand. AutoDock was performed many times to obtain several docked poses. After docking, the best poses leading to the stabilization of the protein–ligand complex with the highest docking score were selected and further investigated. The best pose was selected on the basis of the scoring function estimating the protein–ligand binding energy. In the final post-docking step, the residues (side chains) closest to the active ligand were minimized with respect to the pose found, the ligand was energy-minimized using standard potentials, and the positions of the protons in the entire complex were optimized. The docking results were further verified (redocking task) using a Genetic Evolutionary Method for molecular DOCKing (GEMDOCK) [110], which, similar to AutoDock, uses the genetic algorithm but a different evolution operator (limited Gaussian and Cauchy mutations and introduced rotamer-based mutations). The docking score is defined as a combination of the pairwise inter- and intramolecular energy with penalty term, the energy of binding of pharmacophore summarizing (electrostatic and hydrophilic terms) for all hot-spot atoms and penalty function which limits non-active conformations. Further post-docking exploration of protein–ligand non-covalent and hydrophobic interactions was performed using PoseEdit [111], Discovery Studio Visualizer [112], LigPlot+ [82,83], QTAIM/RDG, and GEMDOCK. Several techniques were employed because detecting weak, non-covalent interactions can be challenging. The GEMDOCK docking score was calculated as a sum of the intramolecular and intermolecular interactions corrected by a penalty value keeping the ligand inside the box (ligand–protein term), the pharmacophore-based interaction between the ligand and protein (binding site term) and penalty value for the electrostatic and hydrophilic preferences of the ligand (penalty term). The energy of the intramolecular interactions was described using the sum of the piecewise linear potential (PLP) term, which describes steric (van der Waals), hydrogen bonding interactions and the Coulomb electrostatic term. The energy of intramolecular interactions was estimated using the Gehlhaar model [113] with original parameterization, which employs piecewise linear potentials and a steric term. The binding affinity was estimated using a multiple linear regression model. The final 3D visualization of the binding modes was made using VMD [107].

### 3.5. Comparison of the Differences in the Binding Modes—Mathematical Metrics

A similarity of the molecules was calculated using the Tanimoto distance, defined as the ratio of the intersection of *A* and *B* to the union of *A* and *B*:

$$d(A,B)=\frac{\left|A\cap B\right|}{\left|A\right|+\left|B\right|-\left|A\cap B\right|}$$

where *A* and *B* are sets characterizing molecular structures.

A similarity of the binding modes was calculated using a few metrics defined below:

- Euclidian distance $d\left(p,q\right)=\sum_{i}\sqrt{{\left(p_{i}-q_{i}\right)}^{2}}$;
- Manhattan distance $d\left(p,q\right)=\sum_{i}\left|p_{i}-q_{i}\right|$;
- the additive formula $d\left(p,q\right)=\sum_{i}\left(p_{i}-q_{i}\right)$;
- cosine distance $d\left(p,q\right)=\frac{\sum_{i}\left(p_{i}q_{i}\right)}{\sum_{i}\left(p_{i}\right)\sum_{i}\left(q_{i}\right)}$;
- Tanimoto distance $d(p,q)=\frac{\left|P\cap Q\right|}{\left|P\right|+\left|Q\right|-\left|P\cap Q\right|}$;

where *p_i_* and *q_i_* are the binding interactions in each structure, P = {*p_i_*}, and Q = {*q_i_*}.

The plots were generated using OriginLab 2024 (OriginLab corporation, Northampton, MA, USA).

## 4. Conclusions

One of the primary objectives of small molecule drug discovery is to find innovative chemical compounds that possess desired properties. For this purpose, new global quantum chemical indices describing relative reactivity and new quantitative methods for the estimation and visualization of the differences in the binding modes of individual analogues with RdRp have been developed and applied, allowing the candidate ligands to be conveniently screened. The use of new relative reactivity indices can aid in the preliminary screening of candidate ligands. Euclidean, Manhattan, and cosine distances are helpful in quantifying the differences in binding modes between native and candidate ligands. Isosurfaces and radial plots can be used to visualize these differences, which is helpful in recognizing their location. This combined approach is particularly useful when comparing modifications or screening candidate drugs.

Quantitative analysis of the binding modes provided important clues to design high-affinity RdRp ligands. The proposed modification of the FVP structure seems to improve its binding ability to the SARS-CoV-2 RdRp and enhance productive binding mode.

In particular, two of the candidate ligands (the trifluoro- and cyano- derivatives) bind very strongly to both the RNA template and RdRp, so they may constitute a very good alternative to FVP. The CN analogue, like FVP, shows low and negative lipophilicity, while the CF_3_ analogue shows high and positive lipophilicity, which is more optimal for drugs. Replacing –F with –CF_3_, which has a strong electron-withdrawing nature, poor polarizability, and a broad hydrophobic domain, results in an increase in the hydrophobicity (lipophilicity) of the ligand and thus modifies the hydrophobic targets. Both CN and CF_3_ analogues seem promising because, as highly electrophilic reagents, they should have low substrate selectivity and inhibit a wide range of RdRps, not only SARS-CoV-2. Moreover, their relative electro-donating and electro-accepting powers are high; thus, both should act more efficiently than FVP-RTP. The CF_3_ analogue appears to be a better choice because it does not affect the preferred arrangement of the amide and triphosphate groups required for the effective binding to RNA primer and Mg^2+^, respectively. However, the CN analogue has a disadvantage because it is conformationally more labile. The CF_3_ analogue incorporated into the RNA strand binds to the RNA template and RNA primer more strongly than FVP, even in monophosphate form. Moreover, it shows the strongest binding to LYS798 among the FVP-RMP analogues and forms a strong binding to the cofactor. On the other hand, the CF_3_ analogue binds directly to RdRp much more weakly than the F or CN derivatives. This direct binding of the CF_3_ analogue is also much weaker than when it is incorporated into the RNA strand.

Upon comparison of all protein–ligand complexes described above, it is apparent that the main channel proteins (LYS545, ARG553, and ARG555) are relatively loosely bound to the ligand in the non-productive and active states. Nevertheless, in the pre-catalytic states and in instances of direct binding, the binding is significantly stronger. The ligand binding to LYS 798 firmly stabilizes the RdRp core in pre-catalytic states, but the binding is feeble in non-productive and direct binding states. The CN analogue exhibits lower selectivity, as it promotes comparable binding modes in pre-catalytic state I and II. Conversely, the CF_3_ analogue primarily reinforces pre-catalytic state I. The latter combined with a better ADME profile, increases CF_3_ attractiveness in terms of selective anti-virial activity. Replacing F with CF_3_ may help to overcome the limitations caused by the alternative mechanism of binding responsible for the scattering of the results of clinical trials or the synergistic effect observed in combined treatment against SARS-CoV-2. Increasing the number of acceptors seems to be a good step toward greater efficiency of FVP analogues. The docking results suggest that among the analogues studied, the CF_3_ derivative should bind most strongly to the cofactor Mg^2+^, the RNA template, and the RNA primer and therefore may be a very good alternative to FVP-RTP and RVD. Additionally, the incorporation of the CF_3_ group is expected to considerably reduce the likelihood of the formation of an alternate metabolite, in which the OH group is attached to the C(5) position due to the steric hindrance.

Our method for quantifying differences in binding mode holds promise for guiding future research on new anti-SARS-CoV-2 agents. If sufficient effectiveness in inhibiting viral replication in cell culture is established, they could be explored as potential drugs against COVID-19.

## Figures and Tables

**Table 1:** The physicochemical profile parameters that describe the pharmacokinetic behavior of the candidate ligands.

R	MW	LogP *	LogP **	Solubility (SILICOS-IT)	Abbot’s Bioavailability	TPSA [A^2^]	SAS	GI	Lead-likeness (Lipinski, Egan, Veber)
H	139.11	−0.65	−1.539	Soluble	0.55	88.84	1.73	High	Yes
F (FVP)	157.1	−0.27	−0.934	Soluble	0.55	88.84	2.08	High	Yes
Cl	173.56	−0.05	−0.472	Soluble	0.55	88.84	2.07	High	Yes
Br	218.01	0.04	−0.293	Soluble	0.55	88.84	2.12	High	Yes
I	265.01	0.06	−0.313	Soluble	0.55	88.84	2.43	High	Yes
CF_3_	207.11	0.52	−0.142	Soluble	0.55	88.84	2.03	High	Yes
CH_3_	153.14	−0.31	−0.982	Soluble	0.55	88.84	1.88	High	Yes
CN	164.12	−0.83	−0.878	Soluble	0.55	112.63	2.10	High	Yes
MOL	329.31	−0.89	−0.402	Soluble	0.55	143.14	4.49	Low	No; 1 violation (MW > 300)
RVD	602.23	0.52	1.961	Moderately soluble	0.17	203.55	6.43	Low	No; 2 violations (MW > 350, rotors > 7, TPSA > 140)
NMVr	499.24	1.64	2.013	Moderately soluble	0.55	131.4	4.82	High	No; 1 violation (rotors > 10, MW > 350)
RTV	720.94	5.04	4.5	Insoluble	0.17	202.26	6.45	Low	No, 3 violations (MW > 350, XLOGP3 > 3.5, rotors > 7)
Optimal values	<500	1 < logP < 4		Soluble	1.0	<140	<10	High	Yes

* SwissADME, ** ADMET2.0.

**Table 2:** The parameters describing the toxicity of the candidate ligands.

R	BBB, PAINS	BRENK	P-gp	CYP1A2, CYP2C19, CYP2C9, CYP2D6, CYP3A4	Carcinogenicity	Genotoxic Carcinogenicity/Mutagenicity	AMES	LD_50_ Rat	H-HT	DILI
H	No	No	No	No	0.134	0	0.022	0.269	0.177	0.956
F (FVP)	No	No	No	No	0.23	0	0.031	0.522	0.833	0.935
Cl	No	No	No	No	0.501	0	0.027	0.636	0.21	0.92
Br	No	No	No	No	0.708	0	0.055	0.962	0.082	0.835
I	No	No	No	No	0.169	0	0.032	0.487	0.101	0.603
CF_3_	No	No	No	No	0.14	0	0.326	0.967	0.491	0.948
CH_3_	No	No	No	No	0.158	0	0.018	0.364	0.331	0.893
CN	No	No	No	No	0.168	0	0.028	0.434	0.893	0.941
MOL	No	No	No	No	0.303	4 rules violation	0.512	0.021	0.486	0.976
RVD	No	1 alert (phosphorus)	Yes	No CYP3A4 Yes	0.314	5 rules violation	0.822	0.719	0.257	0.946
NMVr	No	No	Yes	No CYP3A4 Yes	0.119	0	0.003	0.962	0.293	0.556
RTV	No	No	Yes	No CYP3A4 Yes	0.021	1 rule violation	0.023	0.245	0.965	0.978
Optimal values	No	No	Yes	No	0	0	0	0	0	0

**Table 3:** The global reactivity descriptors calculated at the MP2/6-311G(d,p) level of the theory.

R *	HOMO [eV]	LUMO [eV]	Gap [eV]	Χ [eV]	η [eV]	ω [eV]	S [1/eV]	ω^+^ [eV]	ω^−^ [eV]	Δω [eV]	ΔN_max_ [−]	R^+^ [−]	R^−^ [−]
single molecule (gas)													
H	−9.703	1.672	11.375	4.016	5.688	1.418	0.176	4.136	0.121	4.257	0.706	0.567	0.914
F	−9.864	1.237	11.101	4.313	5.550	1.676	0.180	4.526	0.213	4.740	0.777	1.000	1.000
Cl	−9.763	1.245	11.007	4.259	5.504	1.648	0.182	4.465	0.206	4.672	0.774	0.968	0.987
Br	−9.641	1.270	10.911	4.186	5.455	1.606	0.183	4.380	0.195	4.575	0.767	0.914	0.968
I	−9.465	1.218	10.683	4.124	5.341	1.592	0.187	4.321	0.198	4.519	0.772	0.927	0.955
CF_3_	−10.366	1.138	11.505	4.614	5.752	1.851	0.174	4.877	0.263	5.139	0.802	1.232	1.077
CH_3_	−9.366	1.723	11.090	3.822	5.545	1.317	0.180	3.921	0.099	4.020	0.689	0.466	0.866
CN	−10.201	1.008	11.209	4.597	5.604	1.885	0.178	4.884	0.287	5.171	0.820	1.348	1.079
aqueous solution, pH = 7													
H	−9.582	1.671	11.254	3.955	5.627	1.390	0.178	4.071	0.116	4.187	0.703	0.610	0.929
F	−9.686	1.303	10.989	4.192	5.494	1.599	0.182	4.382	0.190	4.572	0.763	1.000	1.000
Cl	−9.626	1.345	10.971	4.140	5.486	1.562	0.182	4.318	0.178	4.496	0.755	0.937	0.986
Br	−9.544	1.358	10.902	4.093	5.451	1.537	0.183	4.265	0.172	4.436	0.751	0.904	0.973
I	−9.315	1.390	10.705	3.962	5.352	1.467	0.187	4.117	0.155	4.271	0.740	0.814	0.940
CF_3_	−10.104	1.326	11.430	4.389	5.715	1.685	0.175	4.594	0.205	4.800	0.768	1.081	1.049
CH_3_	−9.291	1.674	10.965	3.809	5.482	1.323	0.182	3.912	0.104	4.016	0.695	0.547	0.893
CN	−9.937	1.281	11.169	4.328	5.609	1.670	0.178	4.535	0.207	4.742	0.772	1.089	1.035

* R—substituent.

**Table 4:** The global reactivity descriptors calculated at the M062X/6-311G(d,p) level of the theory.

R *	HOMO [eV]	LUMO [eV]	Gap [eV]	Χ [eV]	η [eV]	ω [eV]	S [1/eV]	ω^+^ [eV]	ω^−^ [eV]	Δω [eV]	ΔN_max_ [−]	R^+^ [−]	R^−^ [−]
single molecule (gas)													
H	−8.750	−1.080	7.669	4.915	3.835	3.150	0.261	6.087	1.172	7.258	1.282	0.805	0.926
F	−8.799	−1.432	7.367	5.115	3.684	3.552	0.271	6.570	1.455	8.025	1.389	1.000	1.000
Cl	−8.722	−1.477	7.246	5.100	3.623	3.589	0.276	6.592	1.492	8.084	1.408	1.026	1.003
Br	−8.574	−1.463	7.112	5.018	3.556	3.541	0.281	6.495	1.477	7.972	1.411	1.015	0.989
I	−8.422	−1.501	6.920	4.961	3.460	3.557	0.289	6.470	1.509	7.979	1.434	1.037	0.985
CF_3_	−9.258	−1.582	7.676	5.420	3.838	3.827	0.261	7.017	1.597	8.614	1.412	1.098	1.068
CH_3_	−8.434	−0.997	7.437	4.715	3.718	2.990	0.269	5.812	1.097	6.909	1.268	0.754	0.885
CN	−9.188	−1.711	7.478	5.449	3.739	3.971	0.267	7.163	1.714	8.877	1.457	1.178	1.090
aqueous solution, pH = 7													
H	−8.675	−1.099	7.576	4.887	3.788	3.152	0.264	6.069	1.182	7.252	1.290	0.830	0.940
F	−8.668	−1.400	7.268	5.034	3.634	3.486	0.275	6.458	1.424	7.881	1.385	1.000	1.000
Cl	−8.634	−1.413	7.220	5.023	3.610	3.495	0.277	6.458	1.435	7.893	1.391	1.008	1.000
Br	−8.533	−1.410	7.122	4.972	3.561	3.470	0.281	6.401	1.430	7.831	1.396	1.004	0.991
I	−8.343	−1.380	6.963	4.861	3.482	3.394	0.287	6.260	1.398	7.658	1.396	0.982	0.969
CF_3_	−9.098	−1.443	7.655	5.271	3.828	3.629	0.261	6.743	1.472	8.215	1.377	1.034	1.044
CH_3_	−8.402	−1.069	7.333	4.735	3.666	3.058	0.273	5.884	1.149	7.033	1.292	0.807	0.911
CN	−8.977	−1.492	7.485	5.235	3.743	3.661	0.267	6.746	1.511	8.257	1.399	1.061	1.045

* R—substituent.

**Table 5:** The degree of interaction, DOI, for FVP and its two analogues, CN and CF_3_.

	FVP		CF_3_		CN	
1	N(4)	3.531	N(4)	3.545	N(4)	3.537
2	C(2)	3.964	C(2)	4.279	C(2)	4.251
3	C(6)	5.151	C(6)	5.715	C(6)	5.897
4	N(1)	3.695	N(1)	3.783	N(1)	3.711
5	C(2)	5.124	C(2)	5.266	C(2)	5.048
6	C(7)	5.294	C(7)	5.453	C(7)	5.390
7	N(7)	3.037	N(7)	3.034	N(7)	3.049
8	=O	2.293	=O	2.291	=O	2.284
9	C(3)	5.154	C(3)	5.378	C(3)	5.277
10	OH	2.492	OH	2.503	OH	2.502
11	F	1.495	C	5.128	C	5.164
11a	-	-	F	1.555	N	2.684
11b	-	-	F	1.511	-	-
11c	-	-	F	1.510	-	-
12	H	0.795	H	0.810	H	0.796
13	H	0.858	H	0.854	H	0.852
14	H(OH)	0.896	H(OH)	0.895	H(OH)	0.899
15	H	1.013	H	1.007	H	0.998

**Table 6:** The list of hydrogen bonds binding FVP-RTP to RdRp in the 7CTT [81] complex.

No.	Residue Chain	Residue Name	H⋯A Distance [Å]	D⋯A Distance [Å]	<DHA	Type	Donor	Acceptor	Moiety
1	621A	LYS	3.30	4.05	133.53	D	4327 [Nam]	8910 [O_3_]	Phosphate
2	622A	CYS	3.03	3.63	120.57	D	4336 [Nam]	8913 [O_2_]	Phosphate
3	623A	ASP	2.60	3.46	147.96	A	8902 [O_3_]	4349 [O_2_]	Ribosyl
4	623A	ASP	2.70	3.59	153.00	A	8903 [O_3_]	4349 [O_2_]	Ribosyl
5	623A	ASP	2.82	3.74	156.06	D	4342 [Nam]	8913 [O_2_]	Phosphate
6	682A	SER	2.09	2.99	150.84	A	8898 [Nam]	4792 [O_2_]	Pyridazine
7	691A	ASN	2.72	3.19	109.79	D	4855 [Nam]	8902 [O_3_]	Ribosyl
8	760A	ASP	3.00	3.77	140.59	D	5420 [O_3_]	8901 [O_3_]	Ribosyl

**Table 7:** The list of salt bridges (opposite charges), π-cation interactions (a positive charge and an aromatic ring), and metallic interactions in the 7CTT [81] complex.

No.	Residue Chain	Residue Name	Distance [Å]	Ligand Moiety	Interaction Type
1	555A	ARG	4.12	Aromatic	π-cation
2	553A	ARG	5.42	Phosphate	ionic/salt bridge
3	553A	ARG	4.78	Phosphate	ionic/salt bridge
4	555A	ARG	5.05	Phosphate	ionic/salt bridge
5	621A	LYS	4.36	Phosphate	ionic/salt bridge
6	798A	LYS	5.32	Phosphate	ionic/salt bridge
7	798A	LYS	5.44	Phosphate	ionic/salt bridge
8	798A	LYS	3.73	Phosphate	ionic/salt bridge
9		Mg^2+^	6.94	Phosphate	metal

**Table 8:** The docking results for FVR-RTP analogues (the RdRp target from 7CTT [81]).

Parameter	FVP *	F	Cl	Br	I	H	CH_3_	CF_3_	CN
binding score **, kcal/mol	-	−6.92	−6.95	−6.94	−6.90	−6.54	−6.96	−10.51	−11.58
docking score, kcal/mol	-	−125.466	−126.032	−125.752	−125.343	−118.565	−126.244	−190.616	−209.746
protein–ligand, kcal/mol	−112.71	−132.906	−134.130	−134.054	−134.117	−130.160	−134.035	−135.452	−167.177
protein–ligand hydrogen bonds, kcal/mol	−13.545	−11.008	−11.438	−11.455	−11.444	−11.005	−11.453	−13.023	−12.502
cofactor-RNA template/primer–ligand, kcal/mol	−54.877	−56.192	−54.984	−54.854	−54.579	−54.348	−59.124	−60.590	−55.773
binding affinity, kJ/mol	−24.66	−29.05	−25.31	−25.31	−25.30	−25.60	−25.26	−76.80	−58.57

* The actual ligand without redocking; ** AutoDock result.

**Table 9:** The ordering of the ligands according to the different parameters describing the binding strength.

Parameter	Ordering
docking score	CN > CF_3_ > CH_3_ > Cl > Br > I > H > F
total energy of binding	CN > CF_3_ > Cl > I > CH_3_ > Br > H > F
hydrogen bonds linking the ligand to the protein	CF_3_ > CN > Br > CH_3_ > I > Cl > F > H
binding of the ligand to the RNA template	CF_3_ > H > F > CN > Br > Cl > I > CH_3_
binding of the ligand to the RNA primer	CF_3_ > F > CH_3_ > Cl > H > Br > I > CN
binding of the ligand to both the RNA template and RNA primer	CF_3_ > F > CH_3_ > Cl > Br > H > I > CN
binding of the ligand to magnesium ion	CN > CF_3_ > H ≅ F > Br > I > CH_3_ > Cl
binding affinity	CF_3_ > CN > F > H > Cl ≅ Br > I > CH_3_

**Table 10:** Comparison of the similarity of binding patterns of the individual ligands with respect to the FPV-RTP ligand (Pre-Catalytic State—Productive Mode I, all residues).

R	The Entire Complex			Protein Residues			Mg^2+^, Zn^2+^, RNA Template/Primer		
	Euclidian	Manhattan	Additive	Euclidian	Manhattan	Additive	Euclidian	Manhattan	Additive
H	1.567	0.647	0.240	1.578	0.627	0.242	1.407	0.934	0.203
Cl	1.581	0.636	0.285	1.551	0.934	0.203	1.551	0.588	0.289
Br	1.576	0.635	0.283	1.549	0.589	0.289	1.919	1.294	0.199
I	1.566	0.631	0.278	1.549	0.590	0.288	1.791	1.228	0.131
CF_3_	1.877	0.756	0.378	1.718	0.691	0.310	3.426	1.696	1.370
CH_3_	1.590	0.640	0.288	1.550	0.588	0.288	2.091	1.384	0.284
CN	2.845	1.008	0.661	2.922	1.008	0.731	1.286	1.005	−0.352
FVP-RTP *	1.585	0.636	0.299	1.529	0.587	0.279	2.234	1.336	0.585

* Redocked vs. actual.

**Table 11:** Comparison of the similarity of binding patterns of the individual ligands with respect to the FPV-RTF ligand (Pre-Catalytic State—Productive Mode I, selected residues).

R	The Entire Complex (Selected Residues, Mg^2+^, Zn^2+^, RNA Template, and RNA Primer)			Selected Residues (555, 798, 621, 553, 682, 545, 691, 622, 623, 760, 761, and 618)		
	Euclidian	Manhattan	Additive	Euclidian	Manhattan	Additive
H	1.492	0.480	0.213	1.498	0.449	0.214
Cl	1.523	0.473	0.273	1.488	0.934	0.203
Br	1.517	0.472	0.269	1.485	0.415	0.274
I	1.507	0.469	0.265	1.486	0.416	0.274
CF_3_	1.803	0.577	0.377	1.632	0.500	0.308
CH_3_	1.533	0.477	0.276	1.486	0.414	0.275
CN	2.729	0.737	0.634	2.801	0.719	0.703
FVP-RTP *	1.512	0.470	0.274	1.448	0.409	0.252

* Redocked vs. actual.

**Table 12:** The binding mode of the FVR-RTP analogues to RdRp (the RdRp target from 7CTT [81]); kcal/mol units. (The active site residues, RNA primer, RNA template, and cofactor are taken into account).

Residue	FVP-RTP *	F	Cl	Br	I	H	CH_3_	CF_3_	CN
RNA primer	−41.019	−45.808	−44.858	−44.732	−44.396	−43.848	−45.166	−48.652	−42.647
RNA template	−13.292	−11.988	−11.262	−11.263	−11.257	−12.039	−11.254	−16.309	−11.66
LYS545	−5.379	−5.136	−4.984	−4.986	−4.991	−4.686	−4.981	−9.728	−5.070
ARG553	−15.047	−18.025	−18.809	−18.756	−18.773	−18.002	−18.773	−16.868	−17.259
ARG555	−31.286	−30.126	−30.431	−30.376	−30.360	−27.792	−30.436	−32.469	−31.030
ASP618	14.767	17.189	15.055	15.158	15.186	17.225	15.116	11.267	4.697
LYS621	−20.662	−30.887	−30.866	−30.872	−30.869	−30.827	−30.880	−31.682	−40.246
CYS622	−4.298	−6.501	−6.469	−6.472	−6.462	−6.520	−6.463	−6.449	−7.593
ASP623	−4.074	−4.861	−5.687	−5.657	−5.637	−4.869	−5.661	−5.553	−7.966
SER682	−6.868	−10.886	−11.051	−11.051	−11.059	−10.859	−11.041	−10.147	−9.894
ASN691	−4.639	−5.911	−6.279	−6.276	−6.266	−5.905	−6.276	−2.873	−6.288
SER759	−0.740	−1.690	−1.857	−1.861	−1.852	−1.686	−1.861	−0.752	−1.061
ASP760	2.617	0.655	1.806	1.776	1.699	0.570	1.786	5.699	2.603
ASP761	4.659	4.242	4.203	4.206	4.204	4.240	4.206	4.363	3.125
LYS798	−23.722	−21.897	−20.299	−20.352	−20.363	−21.910	−20.329	−21.657	−29.618
Mg^2+^	−7.022	−6.450	−6.306	−6.314	−6.313	−6.450	−6.312	−6.464	−5.305
cosine distance (total) **	1.0	0.988	0.985	0.985	0.985	0.985	0.985	0.985	0.956
cosine distance (residues) **	1.0	0.980	0.977	0.977	0.977	0.975	0.977	0.974	0.939

* The actual ligand without redocking ** Cosine distance between the binding mode of FVP-RTP and its analogues.

**Table 13:** The list of most important hydrogen bonds binding RVD to RdRp in 7UO4 [80] complex.

No.	Reside Chain	Residue Name	H⋯A Distance [Å]	D⋯A Distance [Å]	<DHA	Donor/Acceptor	Donor	Acceptor	Moiety
1	621A	LYS	3.17	4.09	155.61	D	4992 [Nam]	12374 [O_3_]	Phosphate
2	622A	CYS	3.12	4.04	156.57	D	5001 [Nam]	12373 [O_3_]	Phosphate
3	623A	ASP	2.64	3.21	117.47	D	5007 [Nam]	12378 [O_3_]	Phosphate
4	623A	ASP	2.15	3.11	168.63	A	12,378 [O_3_]	5014 [O^−^]	Phosphate
5	687A	THR	2.74	3.70	168.20	A	12,379 [O_3_]	5489 [O_3_]	Phosphate
6	691A	ASN	2.00	2.80	136.69	D	5520 [Nam]	12379 [O_3_]	Ribosyl
7	759A	SER	2.91	3.59	128.06	D	6082 [O_3_]	12381 [N1]	Ribosyl

**Table 14:** The list of salt bridges (opposite charges) and metallic interactions in 7UO4 [80].

No.	Residue Chain	Residue Name	Distance [Å]	Ligand Moiety	Interaction Type
1	551A	LYS	5.08	Phosphate	salt bridge
2	798A	LYS	3.58	Phosphate	salt bridge
3		Mg^2+^	2.34	Phosphate	metal
4		Mg^2+^	2.82	Phosphate	metal

**Table 15:** The docking results for FVR-RTP analogues (the RdRp target from 7UO4 [80]).

	RDV *	F	Cl	Br	I	H	CH3	CF3	CN
Tanimoto binding fingerprint distance	1.0	0.735	0.735	0.735	0.735	0.735	0.753	0.753	0.735
binding score **, kcal/mol	-	−8.201	−7.673	−7.663	−7.651	−7.448	−7.292	−7.887	−7.786
docking score, kcal/mol	-	−251.895	−235.698	−235.415	−235.02	−228.801	−223.990	−242.268	−239.205
protein–ligand, total, kcal/mol	−114.144	−156.685	−155.140	−153.131	−155.108	−154.503	−155.543	−158.402	−155.860
protein–ligand hydrogen bonds, kcal/mol	−6.768	−26.465	−24.459	−22.111	−24.378	−25.941	−18.922	−22.176	−23.164
cofactor-RNA template/primer–ligand, total, kcal/mol	−57.037 (−7.328)	−55.375 (−23.783)	−50.282 (−15.983)	−53.411 (−14.170)	−53.411 (−14.055)	−45.374 (−15.276)	−59.404 (−15.487)	−53.785 (−10.571)	−54.901 (−15.961)
hydrogen bonds, total, kcal/mol	−13.439	−26.484	−23.114	−22.107	−22.250	−22.756	−18.649	−18.649	−22.189
binding affinity, kJ/mol	-	−112.947	−84.893	−82.002	−83.860	−84.413	−82.009	−70.516	−83.455

* The actual ligand without redocking, 7 = ** AutoDock result.

**Table 16:** The ligands’ ordering according to the decreasing binding strength.

Parameter	Ordering
docking score	F > CF_3_ > CN > Cl > Br > I > CH_3_ > H
total energy of binding	CF_3_ > F > CN > CH_3_ > Cl > I > H > Br
hydrogen bonds linking the ligand to the protein	F > H > Cl > I > CN > CF_3_ > Br > CH_3_
binding of the ligand to the RNA template	F > CF_3_ > CN > Br > I > H > Cl > CH_3_
binding of the ligand to the RNA primer	CF_3_ > F > CN > Br > CH_3_ > Cl > I > H
binding of the ligand to both the RNA template and RNA primer	F > CF_3_ > CN > Br > CH_3_ > Cl > I > H
binding of the ligand to magnesium ion	F > H > Cl > Br > CN > I > CF_3_ > CH_3_
binding affinity	F > Cl > H > I > CN > CH_3_ > Br > CF_3_

**Table 17:** The differences in binding mode of the particular FVP-RTP analogues relative to actual RVD ligand (Pre-Catalytic State—Productive Mode II, all residues).

R	Complex			Protein			Mg^2+^, Zn^2+^, RNA Template/Primer		
	Euclidian	Manhattan	Additive	Euclidian	Manhattan	Additive	Euclidian	Manhattan	Additive
H	3.074	1.254	0.176	2.419	1.047	0.359	8.098	4.489	−2.678
Cl	2.818	1.208	0.239	2.449	1.054	0.370	6.180	3.618	−1.809
Br	2.675	1.154	0.147	2.402	1.023	0.345	5.362	3.192	−2.934
I	2.833	1.201	0.234	2.450	1.047	0.369	6.291	3.615	−1.871
CF_3_	2.439	1.152	0.172	2.071	0.992	0.220	5.643	3.644	−0.568
CH_3_	2.553	1.074	0.060	1.567	0.793	0.401	8.358	5.454	−5.257
CN	2.665	1.169	0.302	2.497	1.063	0.381	4.546	2.814	−0.928
F	2.741	1.260	0.412	2.509	1.087	0.392	5.148	3.956	0.733

**Table 18:** The comparison of the similarity of binding patterns of the individual ligands in relation to the actual RVD ligand (Pre-Catalytic State—Productive Mode II, selected residues).

R	The Entire Complex (Selected Residues, Mg^2+^, Zn^2+^, RNA Template, RNA Primer)			Active Site Residues (621, 553, 555, 798, 622, 551, 682, 691, 620, 687, 759, 545, 623, 760, 761, 618)		
	Euclidian	Manhattan	Additive	Euclidian	Manhattan	Additive
H	3.035	0.982	0.131	2.366	0.757	0.311
Cl	2.774	0.930	0.203	2.396	0.757	0.332
Br	2.632	0.880	0.107	2.351	0.731	0.302
I	2.792	0.929	0.191	2.399	0.756	0.323
CF_3_	2.105	0.770	0.114	2.005	0.685	0.157
CH_3_	2.512	0.796	0.002	1.496	0.498	0.340
CN	2.619	0.889	0.268	2.444	0.765	0.345
F	2.741	1.260	0.412	2.509	1.087	0.392

**Table 19:** The binding mode of the FVP-RTP analogues with RdRp (the RdRp target from 7UO4 [80]); kcal/mol units.

Residue	RDV *	F	Cl	Br	I	H	CH_3_	CF_3_	CN
RNA primer	−46.462	−46.702	−41.536	−42.079	−41.228	−37.102	−41.599	−48.096	−45.449
RNA template	−18.993	−26.878	−19.374	−20.311	−19.535	−19.460	−19.361	−23.264	−19.867
LYS545	−4.088	−3.222	−3.223	−3.367	−3.268	−3.089	−3.158	−3.124	−3.540
LYS551	−7.972	−7.551	−7.516	−7.679	−7.632	−7.848	−9.866	−9.473	−7.655
ARG553	−8.612	−22.002	−22.002	−21.063	−21.955	−21.964	−18.994	−9.599	−22.066
ARG555	−15.802	−28.985	−28.669	−28.606	−28.995	−18.856	−22.904	−23.205	−22.065
ASP618	6.961	6.871	6.727	6.976	6.864	6.875	5.894	6.467	6.764
LYS621	−13.844	−20.934	−21.102	−20.496	−21.156	−28.162	−26.845	−19.236	−20.700
CYS622	−8.369	−7.207	−7.561	−7.608	−7.477	−7.797	−8.526	−10.510	−7.238
ASP623	−0.638	−2.238	−2.176	−1.930	−1.997	−1.692	−3.646	−1.565	−2.476
SER682	−10.710	−14.323	−13.310	−12.666	−13.161	−13.968	−13.635	−13.314	−12.987
THR687	−4.667	−13.176	−13.316	−12.602	−12.985	−12.841	−11.555	−10.987	−13.311
ASP760	2.611	4.829	4.571	4.505	4.561	4.784	1.278	1.987	4.550
ASP761	4.158	3.825	3.804	3.845	3.813	3.822	3.337	4.156	3.804
LYS798	−23.603	−23.419	−23.449	−23.872	−23.432	−23.473	−18.078	−22.486	−23.559
Mg^2+^	−44.712	−45.197	−45.136	−45.079	−44.971	−45.138	−27.898	−44.230	−45.109
cosine distance (total) **	1.0	0.968	0.964	0.968	0.963	0.956	0.936	0.990	0.976
cosine distance (residues) **	1.0	0.940	0.940	0.947	0.941	0.934	0.925	0.976	0.946
cosine distance to 7CTT	0.628	0.562	0.551	0.550	0.546	0.559	0.610	0.594	0.602

* Actual ligand without redocking ** Cosine distance between the binding mode of RDV and the FVP-RTP analogues.

**Table 20:** The docking results for the FVR-RTP analogues (the RdRp target from 7AAP [84]).

Parameter	FVP−RTP *	F	Cl	Br	I	H	CH_3_	CF_3_	CN
binding score **, kcal/mol	−8.85	−10.40	−9.85	−10.49	−10.80	−10.05	−9.84	−11.74	−10.81
docking score, kcal/mol	−161.033	−189.604	−179.185	−190.741	−196.385	−182.759	−179.006	−213.435	−196.565
protein–ligand, total, kcal/mol	−44.038	−42.534	−46.801	−50.122	−46.242	−44.699	−46.246	−58.546	−44.98
protein–ligand, hydrogen bonds, kcal/mol	−5.089	−4.152	−7.057	−7.162	−4.529	−7.481	−7.829	−8.902	−5.461
cofactor-RNA template/primer–ligand, total, kcal/mol	−66.056	−39.391	−57.662	−47.412	−21.649	−37.123	−58.932	−57.830	−30.120
binding affinity, kJ/mol	−25.460	−79.409	−79.795	−85.965	−83.816	−82.637	−76.086	−100.253	−72.851

* The actual ligand without redocking; ** AutoDock result.

**Table 21:** The differences in binding mode of the particular FVP-RTP analogues relative to actual ligand FVP-RTP (Pre-Catalytic State—Non-Productive Mode, all residues).

R	Complex			Protein Residues			Mg^2+^, Zn^2+^, RNA Template/Primer		
	Euclidian	Manhattan	Additive	Euclidian	Manhattan	Additive	Euclidian	Manhattan	Additive
H	2.273	0.589	0.223	0.597	0.242	−0.025	7.762	4.626	3.045
Cl	1.887	0.490	0.217	0.574	0.207	0.022	6.370	3.778	2.449
Br	2.012	0.609	0.529	0.631	0.279	0.279	6.774	4.455	3.460
I	3.446	0.868	0.478	0.645	0.309	−0.004	11.955	7.341	5.985
CF_3_	4.076	1.065	0.671	1.042	0.526	0.160	13.941	7.383	6.557
CH_3_	1.542	0.435	0.192	0.591	0.208	0.027	5.067	3.081	2.091
CN	3.007	0.801	0.330	1.116	0.337	−0.077	9.927	6.206	4.956
F	2.643	0.677	0.307	0.555	0.248	−0.027	9.131	5.648	4.112

**Table 22:** Comparison of the similarity of binding patterns of individual ligands in relation to the current FVP-RTP ligand (Pre-Catalytic State—Non-Productive Mode, selected residues).

R	The Entire Complex (Selected Residues, Mg^2+^, Zn^2+^, RNA Template, RNA Primer)			Active Site Residues (545, 682, 555, 553, 814, 551, 691, 798, 687, 622, 760, 623, 618, 761)		
	Euclidian	Manhattan	Additive	Euclidian	Manhattan	Additive
H	2.271	0.545	0.221	0.588	0.193	−0.027
Cl	1.885	0.452	0.222	0.568	0.165	0.028
Br	2.008	0.541	0.461	0.616	0.204	0.204
I	3.439	0.757	0.471	0.605	0.186	−0.012
CF_3_	4.062	0.888	0.647	0.980	0.327	0.134
CH_3_	1.541	0.409	0.192	0.587	0.180	0.026
CN	3.004	0.719	0.328	1.105	0.246	−0.079
F	2.639	0.601	0.302	0.535	0.163	−0.032

**Table 23:** The binding affinity of the FVP-RTP analogues to RdRp (the RdRp target from 7AAP); kcal/mol units.

Parameter	FVP-RTP *	F	Cl	Br	I	H	CH_3_	CF_3_	CN
RNA primer	−36.368	−37.596	−36.257	−39.591	−42.655	−37.085	−35.759	−44.212	−39.227
RNA template	−26.551	−21.173	−22.008	−23.071	−21.805	−21.016	−23.694	−23.658	−22.178
LYS545	−9.973	−9.094	−11.129	−12.049	−8.987	−12.001	−11.715	−13.715	−10.082
LYS551	−4.067	−3.951	−3.622	−3.900	−3.746	−3.623	−3.796	−3.826	−4.172
ARG553	−4.621	−4.323	−3.967	−5.312	−4.452	−4.059	−4.527	−5.341	−4.098
ARG555	−8.070	−6.249	−7.762	−6.433	−6.640	−5.964	−6.792	−9.788	−8.180
ASP618	5.238	5.331	5.281	5.637	3.616	5.575	5.517	4.344	5.941
LYS621	−2.203	−2.357	−2.096	−2.537	−2.465	−2.199	−2.298	−2.864	−2.408
CYS622	−0.565	−0.819	−0.771	−0.734	−0.822	−0.818	−0.581	−0.960	−0.995
ASP623	1.811	0.812	−2.265	−1.551	1.544	1.209	−1.191	−0.339	1.148
SER682	−8.537	−8.580	−7.740	−7.216	−9.875	−8.078	−8.172	−6.880	−8.805
THR687	−2.717	−1.879	−1.163	−0.515	−2.183	0	0	−1.189	−0.872
ASP760	1.789	0.333	0.878	0.653	0.741	−0.843	0	0.301	−0.847
ASP761	6.171	6.377	6.719	6.267	6.348	6.752	6.450	6.198	6.789
LYS798	−3.326	−4.516	−3.313	−3.689	−4.602	−3.495	−3.498	−5.348	−4.271
Mg^2+^	−28.566	−47.517	−35.764	−40.192	−56.691	−36.563	−35.608	−64.101	−44.510
Mg^2+^	−0.308	−0.325	−0.313	−0.318	−0.329	−0.317	−0.314	−0.345	−0.327
Mg^2+^	−44.103	−58.033	−58.648	−56.893	−56.244	−62.176	−55.132	−49.372	−64.308
cosine distance (total) **	0.973	0.983	0.985	0.963	0.978	0.987	0.945	0.977	0.973
cosine distance (residues) **	0.945	0.939	0.931	0.943	0.936	0.936	0.923	0.946	0.945

* The actual ligand without redocking, ** Cosine distance between the binding mode of RDV and the FVP-RTP analogues.

**Table 24:** The docking results for FVP-RMP analogues (the RdRp target from 7DFG [85]).

Parameter	FVP-RMP *	CF_3_	CN
binding score **, kcal/mol	−29.20	−35.51	−35.90
docking score, kcal/mol	−529.700	−648.051	−654.057
protein–ligand, total, kcal/mol	−192.623	−219.376	−212.312
steric, total, kcal/mol	−155.733	−155.733	−153.221
van der Waals, total, kcal/mol	−55.040	−61.936	−61.538
protein–ligand hydrogen bonds, total, kcal/mol	−21.747	−26.440	−23.089
cofactors, total, kcal/mol	−1.076	−3.126	−1.662
binding affinity, kJ/mol	−72.457	−75.135	−69.515

* The actual ligand, ** AutoDock.

**Table 25:** The binding mode of the FVP-RMP analogues to RdRp (target from 7DFG [85]); kcal/mol units.

Residue	FVP-RMP *	CF_3_	CN
LYS500	−6.213	−5.970	−6.190
ARG513	−9.294	−4.533	−4.611
LYS545	−6.555	−9.620	−8.610
ARG555	−5.812	−9.247	−7.042
SER682	−13.242	−13.258	−13.578
SER759	−6.137	−5.961	−6.014
CYS813	−16.024	−15.159	−15.478
SER814	−9.724	−11.143	−10.050
ARG836	−32.449	−38.565	−36.865
LYS849	−23.107	−23.623	−23.229
ARG858	−24.517	−28.346	−29.649
SER861	−8.122	−8.890	−10.14
Mg^2+^	−23.746	−23.381	−23.695
Mg^2+^	−12.684	−13.075	−12.743
cosine distance	1.0	0.991	0.993

* The actual ligand without redocking.

**Table 26:** The differences in binding mode of the particular FVP-RTP analogues relative to actual ligand FVP-RTP (Active stat, all residues).

R	Complex			Protein Residues			Mg^2+^, Zn^2+^, RNA Template		
	Euclidian	Manhattan	Additive	Euclidian	Manhattan	Additive	Euclidian	Manhattan	Additive
CF_3_	0.919	0.381	0.140	0.933	0.380	0.166	0.860	0.466	−0.266
CN	0.698	0.311	0.118	0.746	0.325	0.124	0.052	0.138	0.044

**Table 27:** Comparison of the similarity of binding patterns of individual ligands in relation to the current FVP-RTP ligand (Active state, selected residues).

R	The Entire Complex (Selected Residues, Mg^2+^, Zn^2+^, RNA Template)			Active Site Residues (836, 858, 849, 813, 682, 814, 513, 861, 545, 500, 759, 555, 862)		
	Euclidian	Manhattan	Additive	Euclidian	Manhattan	Additive
CF_3_	0.652	0.184	0.074	0.503	0.126	0.058
CN	0.506	0.146	0.079	0.438	0.113	0.052

**Table 28:** The binding mode of the FVP-RTP analogues to RdRp (the RdRp target from 6M71 [74]).

	F	Cl	Br	I	H	CH_3_	CF_3_	CN
binding score *, kcal/mol	−8.42	−6.00	−6.80	−8.05	−5.60	−6.15	−6.52	−7.97
docking score, kcal/mol	−152.723	−108.945	−123.513	−146.582	−101.65	−111.641	−118.237	−144.458
protein–ligand, total, kcal/mol	−157.797	−121.337	−143.673	−156.914	−127.477	−124.751	−129.888	−149.934
protein–ligand hydrogen bonds, total	−21.207	−39.023	−18.336	−21.327	−19.156	−14.831	−19.482	−20.409
binding affinity, kJ/mol	−34.145	−34.102	−34.983	−34.245	−32.689	−30.110	−40.309	−31.148

* AutoDock.

**Table 29:** The binding mode of the FVP-RTP analogues to RdRp (the RdRp target from 6M71 [74]); kcal/mol units.

Residue	F	Cl	Br	I	H	CH_3_	CF_3_	CN
LYS545	−33.460	−26.092	−26.185	−33.609	−27.282	−24.831	−32.410	−26.185
ARG553	−14.026	−13.621	−9.488	−13.234	−9.836	−13.100	−15.893	−9.488
ARG555	−20.555	−21.398	−23.292	−19.977	−19.827	−22.241	−20.148	−23.292
ASP618	1.069	1.088	1.087	1.061	1.114	1.125	1.110	-
ASP623	−10.604	−6.813	−9.559	−10.063	−4.021	−4.222	−9.262	−9.559
SER682	−17.852	−6.008	−19.222	−17.973	−15.860	−9.322	−0.381	−19.222
THR687	−12.302	−12.053	−12.409	−12.342	−7.133	−10.746	−10.406	−12.409
ASN691	−8.817	−1.631	−9.319	−9.208	−6.982	−3.638	−5.182	−9.319
SER759	−6.907	−8.066	−6.738	−6.907	−6.410	−1.590	−6.602	−6.738
ASP760	−3.664	-	−3.616	−3.616	−3.339	−3.885	−0.814	−3.616
ASP761	1.484	1.549	1.549	1.485	1.572	1.613	1.567	1.550
LYS798	−0.960	−0.965	−0.959	−0.953	−0.969	−0.967	−0.998	−0.951
cosine distance **	1.0	0.953	0.985	1.000	0.987	0.964	0.930	0.985

** Cosine distance calculated relative to the F analogue.

## Data Availability

The data presented in this study are available in article and Supplementary Materials.

## Supplementary Materials

The following supporting information can be downloaded at https://www.mdpi.com/article/10.3390/molecules29020441/s1. Figure S1. The comparison of the total binding energy of FVR-RTP analogues to RdRp, RNA primer, RNA template, and cofactor (the RdRp target from 7CTT [81]); Figure S2. A LigPlot+ schematic 2D representation of the protein–ligand interactions (the RdRp target from 7CTT [81]). The hydrogen bonds are shown as green dashed lines, and residues involved in hydrophobic contacts are shown as red arcs and labeled; Figure S3. The comparison of the total binding affinity of FVR-RTP analogues with RdRp, RNA primer, RNA template, and cofactor (the RdRp target from 7UO4); Figure S4. A LigPlot+ schematic 2D representation of the protein–ligand interactions (the RdRp target from 7UO4 [80]). The hydrogen bonds are shown as green dashed lines, and residues involved in hydrophobic contacts are shown as red arcs and labeled; Figure S5. A LigPlot+ schematic 2D representation of the protein–ligand interactions (target from 7AAP [84]). The hydrogen bonds are shown as green dashed lines, and residues involved in hydrophobic contacts are shown as red arcs and labeled.; Figure S7. A LigPlot+ schematic 2D representation of the protein–ligand interactions (the RdRp target from 6M71 [74]). The hydrogen bonds are shown as green dashed lines, and residues involved in hydrophobic contacts are shown as red arcs and labeled.

## Abbreviations

**Abbreviation**	**Meaning**
ADME	Absorption, Distribution, Metabolism, and Excretion
BBB	blood–brain barrier
CYP	cytochrome P450
DFT	Density Functional Theory
DILI	drug-induced liver injury
DOI	degree of interaction
FVP	favipiravir
GI	gastrointestinal absorption
HGPRT	hypoxanthinguanine phosphoribosyl transferase
H-HT	hepatotoxicity
MD	Molecular Docking
MOL	Molnupiravir
NMVr	Nirmatrelvir
PAINS	Pan Assay of Interference Structures
PCM	polarizable continuum model
P-gp	permeability glycoprotein
QSPR	Quantum Quantitative Structure–Property Relationship
QTAIM	Quantum Theory of Atoms in Molecules
RDG	Reduced Density Gradient
RdRp	RNA-dependent RNA polymerase
RDP	ribonucleoside-5′-diphosphate
RMP	ribonucleoside-5′-monophosphate
RTP	ribonucleoside-5′-triphosphate
RTV	Ritonavir
RNA	ribonucleic acid
RVD	Remdesivir
RBV	Ribavirin
SAS	synthetic accessibility score
ssRNA	single-stranded RNA viruses
TPSA	topological polar surface area
