# Supplementary material for: Favipiravir Analogues as Inhibitors of SARS-CoV-2 RNA-Dependent RNA Polymerase, Combined Quantum Chemical Modeling, Quantitative Structure–Property Relationship, and Molecular Docking Study
Source: Molecules. 2024 Jan 16;29(2):441. doi: 10.3390/molecules29020441 (PMC10818557; doi:10.3390/molecules29020441)
Supplement: Supplementary file 1 [file molecules-29-00441-s001.zip › molecules-2716578-supplementary.pdf]

Favipiravir Analogues as Inhibitors of SARS-CoV-2 RNA-dependent RNA Polymerase, Combined Quantum Chemical Modelling, QSPR and Molecular Docking Study

Magdalena Latosińska and Jolanta Natalia Latosińska*

Supplementary

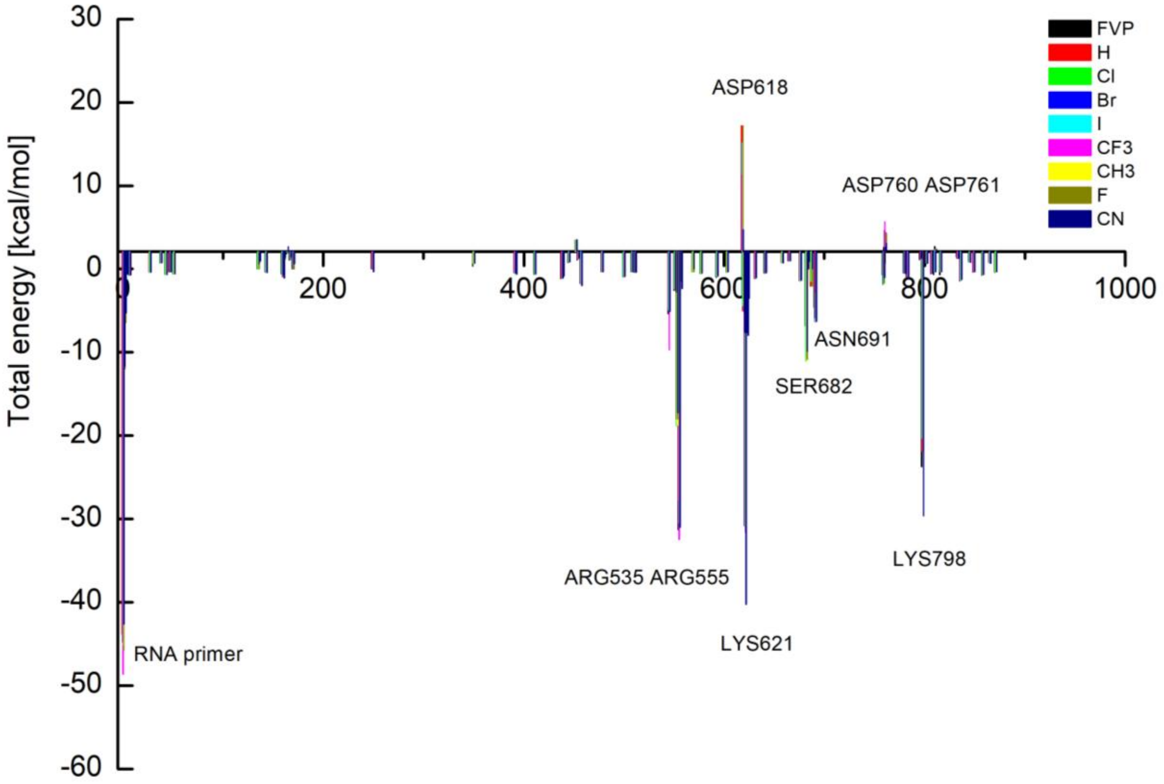

Figure S1. The comparison of the total binding energy of FVR-RTP analogues to RdRp, RNA primer, RNA template and cofactor (the RdRp target from 7CTT [81]).

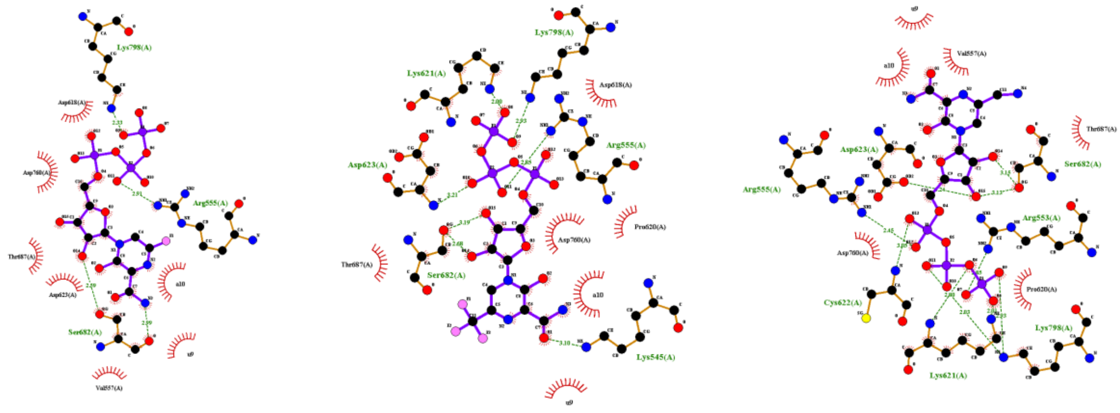

FVP **CF_3** CN

Figure S2. A LigPlot+ schematic 2D representation of the protein-ligand interactions (the RdRp target from 7CTT[81]). The hydrogen bonds are shown as green dashed lines, residues involved in hydrophobic contacts are shown as red arcs and labeled.

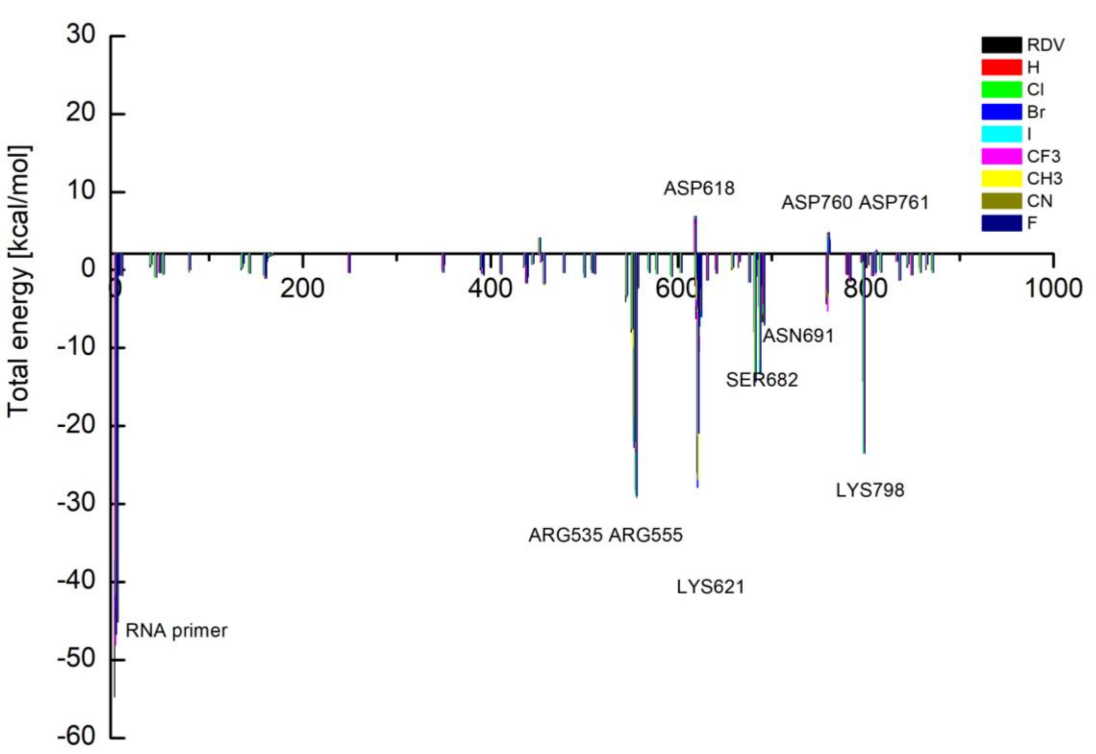

Figure S3. The comparison of the total binding affinity of FVR-RTP analogues with RdRp, RNA primer, RNA template and cofactor (the RdRp target from 7UO4).

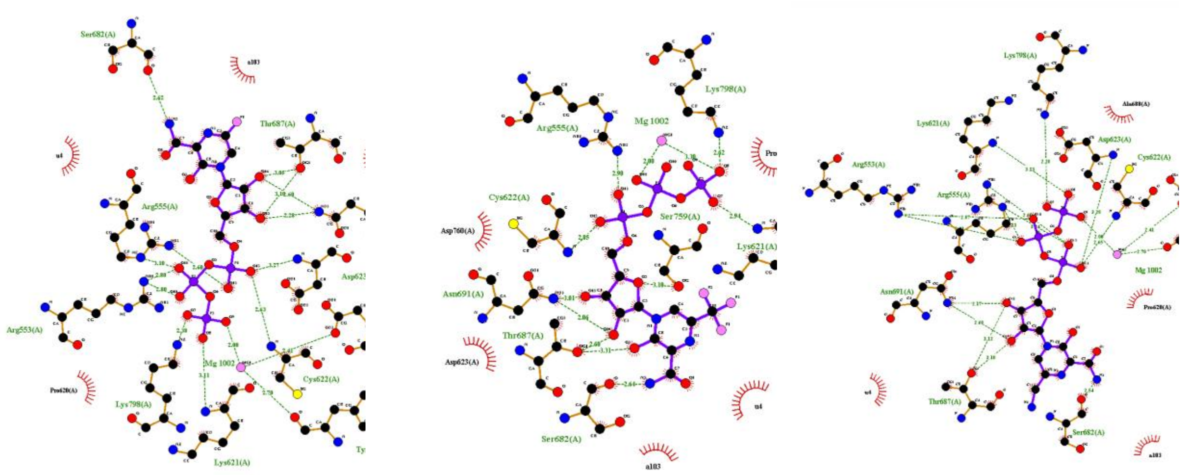

FVP-RTP CF$_3$ CN

Figure S4. A LigPlot+ schematic 2D representation of the protein-ligand interactions (the RdRp target from 7UO4[80]).. The hydrogen bonds are shown as green dashed lines, residues involved in hydrophobic contacts are shown as red arcs and labeled.

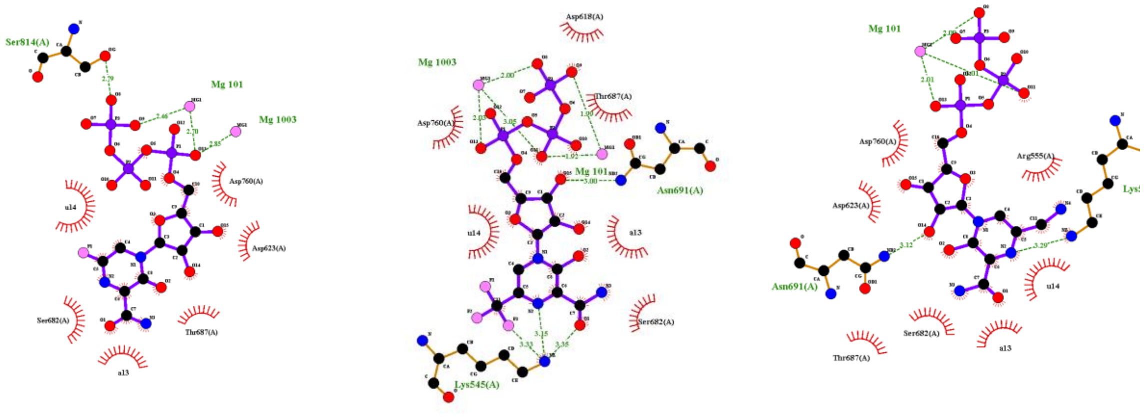

FVP-RTP CF3 CN

Figure S5. A LigPlot+ schematic 2D representation of the protein-ligand interactions (target from 7AAP[84]). The hydrogen bonds are shown as green dashed lines, residues involved in hydrophobic contacts are shown as red arcs and labeled.

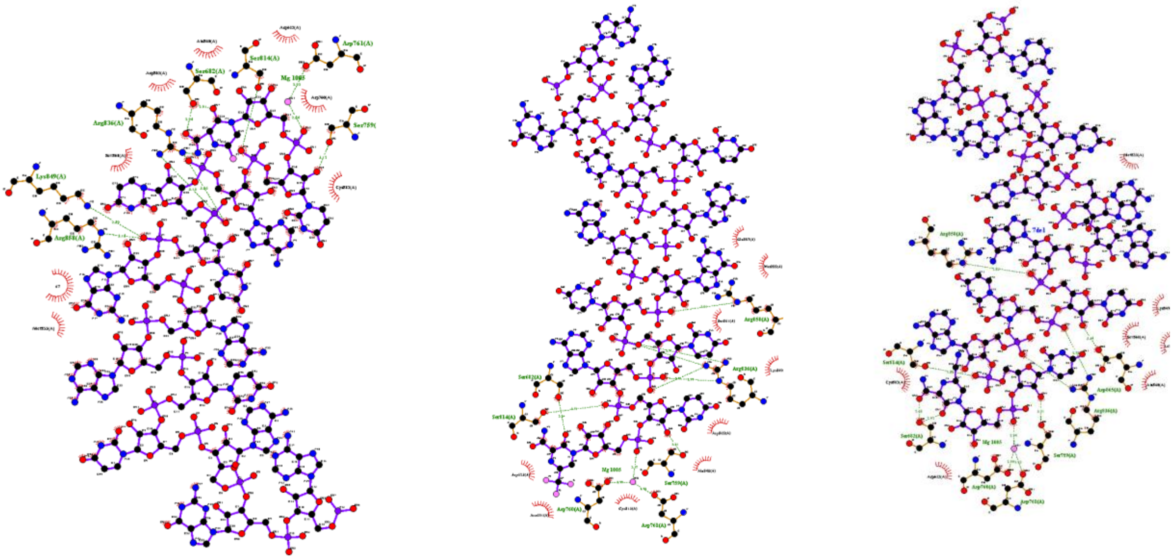

FVP-RTP CF3 CN

Figure S6. A LigPlot+ schematic 2D representation of the protein-ligand interactions (target from 7DFG[85]). The hydrogen bonds are shown as green dashed lines, residues involved in hydrophobic contacts are shown as red arcs and labeled.

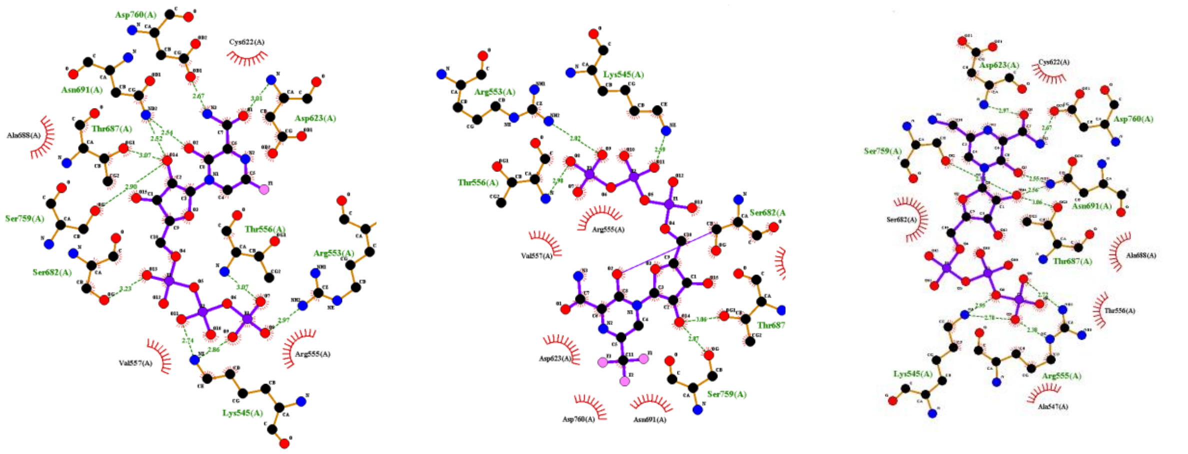

FVP-RTP CF$_3$ CN

Figure S7. A LigPlot+ schematic 2D representation of the protein-ligand interactions (the RdRp target from 6M71[74])). The hydrogen bonds are shown as green dashed lines, residues involved in hydrophobic contacts are shown as red arcs and labeled.
